# Physiological and RNA-Seq Analyses on Exogenous Strigolactones Alleviating Drought by Improving Antioxidation and Photosynthesis in Wheat (*Triticum aestivum* L.)

**DOI:** 10.3390/antiox12101884

**Published:** 2023-10-20

**Authors:** Miao Song, Naiyue Hu, Sumei Zhou, Songxin Xie, Jian Yang, Wenqi Ma, Zhengkai Teng, Wenxian Liang, Chunyan Wang, Mingna Bu, Shuo Zhang, Xiwen Yang, Dexian He

**Affiliations:** 1 College of Agronomy, Henan Agricultural University, Zhengzhou 450046, China; 15003803253@stu.henau.edu.cn (M.S.); hny@henau.edu.cn (N.H.); nxyzsm@henau.edu.cn (S.Z.); xsx175533@stu.henau.edu.cn (S.X.); nxy_yangjian@stu.henau.edu.cn (J.Y.); 17837109363@163.com (W.M.); tzk@stu.henau.edu.cn (Z.T.); wxliang@stu.henau.edu.cn (W.L.); wangchunyan@stu.henau.edu.cn (C.W.); mingnabu@stu.henau.edu.cn (M.B.); nxyzhang@stu.henau.edu.cn (S.Z.); 2 Co-Construction State Key Laboratory of Wheat and Maize Crop Science, Zhengzhou 450046, China; 3 Collaborative Innovation Center of Henan Grain Crops, Zhengzhou 450046, China

**Keywords:** wheat, strigolactones, drought, RNA-seq, antioxidation, photosynthesis, ROS

## Abstract

Drought poses a significant challenge to global wheat production, and the application of exogenous phytohormones offers a convenient approach to enhancing drought tolerance of wheat. However, little is known about the molecular mechanism by which strigolactones (SLs), newly discovered phytohormones, alleviate drought stress in wheat. Therefore, this study is aimed at elucidating the physiological and molecular mechanisms operating in wheat and gaining insights into the specific role of SLs in ameliorating responses to the stress. The results showed that SLs application upregulated the expression of genes associated with the antioxidant defense system (*Fe/Mn-SOD*, *PER1*, *PER22*, *SPC4*, *CAT2*, *APX1*, *APX7*, *GSTU6*, *GST4*, *GOR*, *GRXC1*, and *GRXC15*), chlorophyll biogenesis (*CHLH*, and *CPX*), light-harvesting chlorophyll A-B binding proteins (*WHAB1.6*, and *LHC Ib-21*), electron transfer (*PNSL2*), E3 ubiquitin-protein ligase (*BB*, *CHIP*, and *RHY1A*), heat stress transcription factor (*HSFA1*, *HSFA4D*, and *HSFC2B*), heat shock proteins (*HSP23.2*, *HSP16.9A*, *HSP17.9A*, *HSP21*, *HSP70*, *HSP70-16*, *HSP70-17*, *HSP70-8*, *HSP90-5*, and *HSP90-6*), DnaJ family members (*ATJ1*, *ATJ3*, and *DJA6*), as well as other chaperones (*BAG1*, *CIP73*, *CIPB1*, and *CPN60I*). but the expression level of genes involved in chlorophyll degradation (*SGR*, *NOL*, *PPH*, *PAO*, *TIC55*, and *PTC52*) as well as photorespiration (*AGT2*) was found to be downregulated by SLs priming. As a result, the activities of superoxide dismutase (SOD), peroxidase (POD), and catalase (CAT) were enhanced, and chlorophyll content and photosynthetic rate were increased, which indicated the alleviation of drought stress in wheat. These findings demonstrated that SLs alleviate drought stress by promoting photosynthesis through enhancing chlorophyll levels, and by facilitating ROS scavenging through modulation of the antioxidant system. The study advances understandings of the molecular mechanism underlying SLs-mediated drought alleviation and provides valuable insights for implementing sustainable farming practice under water restriction.

## 1. Introduction

In the context of climate change, drought emerges as a prominent abiotic stress posing a significant threat to global crop production [1]. As a major cereal crop, most of the growing areas of wheat (*Triticum aestivum* L.) are in arid or semi-arid zones affected by climate change. Consequently, the growth of wheat is frequently compromised due to drought stress [2,3]. Drought triggers the generation of reactive oxygen species (ROS), which inflicts oxidative damage on biological macromolecules [4]. Additionally, drought induces chlorophyll degradation and reduces photosynthetic efficiency, resulting in decreased biomass and yield [5]. A meta-analysis revealed that drought stress significantly reduced wheat grain yield and protein yield by 57.32% and 46.04%, respectively [6]. In the backdrop of the projected global population reaching 9 billion by 2050, there will be an increased demand for food, particularly for wheat grain [7]. Therefore, enhancing wheat drought tolerance is crucial to ensure global food security.

To address this issue, various agronomic practices have been explored, including drought priming [8], tillage and cultivation [9], fertilizer management [10], and exogenous regulator application [11]. The application of exogenous phytohormones, such as abscisic acid [12], methyl jasmonate [13], salicylic acid [14], and melatonin [15], has demonstrated potential in enhancing drought tolerance of wheat.

SLs, a class of terpenoid lactones, control several critical processes in plants. Firstly, SLs control plant architecture by inhibiting shoot branching, and promoting internode elongation [16]. Secondly, SLs can also improve nutrient uptake. SLs amplify plant Pi starvation signals by altering metabolic profiles to cope with Pi limitation, and SLs can act as early modulators of plant responses to P starvation [17]. Lastly, SLs positively modulate tolerance to abiotic stresses in plants. SLs improve the alkaline tolerance by regulating the antioxidant defense capacity in soybean [18].

Furthermore, the utilization of exogenous strigolactones (SLs), a novel class of phytohormones, has also been proved to be effective in reducing the damage caused by drought in wheat [14,19,20,21]. Foliar application of SLs has been shown to enhance drought tolerance in wheat by reducing electrolyte leakage and increasing relative water content and photosynthetic pigment content, while also mitigating stomatal limitations on leaves, maintaining the membrane stability index, enhancing antioxidant enzyme activities, decreasing leaf water potential and MDA content, improving photosynthesis, and ultimately increasing the grain yield [14,19,20,21]. Root application of SLs could increase antioxidant enzyme activity, decrease leaf water potential and MDA content, as well as improve photosynthesis and yield [19]. And for wheat plants under drought stress conditions, root application of SLs demonstrated superior efficacy in alleviating drought damages compared with that by foliar application [19].

Previous studies have predominantly focused on foliar SLs for alleviating drought stress in wheat; however, it has been observed that root application of SLs exhibits greater efficacy. Therefore, further research needs to be conducted to explore the potential of root-applied SLs in alleviating drought stress in wheat. Despite the acknowledged enhancement of drought tolerance in wheat through exogenous SLs, there exists a dearth of molecular information concerning the regulatory role of SLs in wheat plants’ response to drought stress. Hence, this study is aimed at examining the phenotypic, physiological, and molecular responses to drought stress after root application of SLs and elucidate the integrated mechanism underlying the ameliorative effect of SLs on wheat under drought stress. These findings may provide valuable insights into putative pathways mediated by SLs and identify regulatory genes capable of ameliorating drought stress and thereby enhancing future wheat production.

## 2. Materials and Methods

### 2.1. Plant Materials and Growth Conditions

Two distinct wheat varieties, Zhoumai 28 (drought-sensitive, V1) and Luohan 22 (drought-tolerant, V2), were selected as plant materials. Twelve seeds of each variety were sown in PVC pots (20 cm × 20 cm × 11.5 cm) filled with soil at 25.0% field capacity. Following emergence, the seedlings were thinned out, retaining only nine plants per pot. The wheat plants were consistently cultivated in a growth chamber under controlled conditions comprising a photoperiod of 16 h light and 8 h dark, with day/night temperatures maintained at 20/18 °C, respectively, along with a relative humidity of 70%. Appropriate soil moisture content was ensured through daily watering practices (Figure S1).

### 2.2. Plant Treatment and Sampling

Each variety was subjected to three treatments: the control group (CK), the drought stress treatment group (T1), and the drought stress with SLs priming group (T2). The experimental design is illustrated in Figure S2. Prior to the drought treatment, T2 plants were treated with 30 mL 10 μM GR24 solution (Solarbio, China, synthetic analogues of SLs) via root irrigation for two consecutive days [19]. Subsequently, T1 and T2 plants at the three-leaf stage were exposed to drought stress by withholding watering. All drought-treated plants were rehydrated after 5 d of drought treatment. Four sampling time points were selected: 0 d, 2 d, 5 d after drought treatment, and 1 d after rehydration treatment. Fresh leaf samples were used for the analysis of leaf water relations analysis, chlorophyll content, and photosynthetic parameters. Additionally, some leaf samples were flash-frozen in liquid nitrogen for RNA-seq analysis, antioxidant activity assessment, as well as H_2_O_2_ and MDA content determination. Certain root samples were examined to determine root phenotype, while other root and shoot samples were dried for measuring dry weight.

### 2.3. Measurement of Dry Weight and Root Phenotype

The plant materials were classified into roots and shoots, and subsequently dried at a constant temperature of 80 °C until reaching a stable weight. To avoid crossing or overlap, the roots were carefully arranged on an acrylic tray filled with water. Subsequently, a root scanner (EPSON EXPRESSION 10000XL, Epson, Beijing, China) was utilized to capture high-resolution images of the roots, which were then analyzed using WinRHIZO Pro2007 software (Regent Instruments Inc., Canada) for precise determination of root length and volume.

### 2.4. Determination of Leaf Water Relations

The upper fully expanded leaves were cut into approximately 1 cm segments. The fresh weight was determined by immersing 0.50 g of the leaves in 20 mL of distilled water, with the tube vacuumed to ensure a complete submersion of the leaves. Subsequently, the leaves were soaked in distilled water at 4 °C for a duration of 24 h, followed by gentle blotting with tissue paper to ascertain turgid weight. Dry weight measurements were obtained after subjecting these samples to constant drying at 80 °C. Leaf relative water content (RWC) and relative saturation deficit (RSD) were calculated using the methods described by Ahmed et al. [22].

### 2.5. Determination of Content of Chlorophyll

The determination was conducted following the method described by Zhang et al. [23]. Shortly, 0.2 g of fresh leaves were placed in 15 mL tubes and chlorophyll was extracted using a mixture of anhydrous ethanol and acetone (*v*/*v* 1:1) through incubation in the dark at room temperature for 24 h. Subsequently, the samples were centrifuged at 10,000× *g* for 15 min and the absorbance was measured at wavelengths of 663 nm and 645 nm using a microplate reader (MADAPA, Shanghai, China). The chlorophyll content was calculated according to Zhang et al. [23]. Additionally, the relative chlorophyll concentrations (SPAD) in the upper fully expanded leaves were determined using a SPAD meter (SPAD-502, Konica-Minolta, Tokyo, Japan).

### 2.6. Measurement of Photosynthetic Parameters

The net photosynthetic rate (Pn), transpiration rate (Tr), stomatal conductance (Gs), and intercellular CO_2_ concentration (Ci) of the upper fully expanded leaves of wheat were assessed using a portable photosynthesis measurement system (Li−6400; LI−COR Inc., Lincoln, NE, USA). The experimental setup followed the methodology outlined by Wang et al. [24].

### 2.7. Determination of Enzymatic Antioxidant Activity and H_2_O_2_ and MDA Content in Leaves

To assess the enzymatic antioxidant activity, 0.25 g of ground leaves were added to 3.0 mL of pre-cooled 50 mM PBS (pH = 7.8) and centrifuged at 12,000× *g* for 20 min at 4 °C. The activities of superoxide dismutase (SOD; EC 1.15.1.1), peroxidase (POD; EC 1.11.1.7), and catalase (CAT; EC 1.11.1.6) were determined following the methodology outlined in a previous study [25].

For the SOD activity assay, 3.0 mL of 50 mM PBS (pH 7.8) containing 9.9 mM L-methionine, 57 μM nitro-blue tetrazolium (NBT), and 0.0044% (*w*/*v*) riboflavin were combined with 0.2 mL of enzyme extract. After a duration of 30 min, the test tube was removed from the light source with an intensity of 4000 lux in order to terminate the reaction. The resulting purple formaldehyde product from NBT was quantified at an absorbance wavelength of 560 nm. In this experiment, one enzyme unit was defined as the volume of supernatant in which the reaction is suppressed by half. For the POD activity assay, 30 μL of leaf enzyme extract was added to a 3 mL reaction mixture containing 0.2 M PBS (pH 6.0), 0.38% guaiacol, and 0.17% H_2_O_2_. One unit of POD enzyme activity was defined as the increase in absorbance at 436 nm by units per minute under the specified assay conditions. For the CAT activity assay, 0.1 mL of leaf enzyme extract was added to a 3 mL reaction mixture containing a 0.15 M PBS (pH 7.0) and 0.46% H_2_O_2_ solution. One unit of CAT enzyme activity represented the change in absorbance at 240 nm by units per minute under the specified assay conditions.

The malondialdehyde (MDA) content, an indicator of lipid peroxidation, was determined using thiobarbituric acid (TBA) colorimetry, as described by Rosemary et al. [25]. Frozen leaf powder (0.5 g) was homogenized with a 5% trichloroacetic acid (TCA) solution (*w*/*v*), followed by centrifugation at 12,000× *g* for 20 min at 4 °C. The reaction mixture comprised 1.5 mL tissue extract and 1.5 mL reagent (0.67% thiobarbituric acid in 5% TCA), which was incubated for 30 min at 100 °C. After cooling the reaction mixture on ice bath, the resulting red pigments were measured at 450 nm, 532 nm, and 600 nm wavelengths. MDA content was calculated using the formula: MDA content = 6.45 × (A532 − A600) − 0.56 × A450.

The hydrogen peroxide (H_2_O_2_) content was quantified using the method described by Sedaghat et al. [19]. H_2_O_2_ was extracted from 0.5 g of leaves homogenate in 0.5 mL of 0.1% (*w*/*v*) trichloroacetic acid (TCA). The homogenates were centrifuged at 12,000× *g* and 4 °C for 10 min; the reaction mixture consisted of 0.5 mL of leaf extract supernatant, 2 mL of reagent solution (1 M KI in double distilled water), and 0.5 mL of a pH 7.0, 10 mM K-phosphate buffer solution. In the absence of leaves extract, the blank control contained only 0.1% TCA solution. The reaction was carried out in darkness for one hour, and the H_2_O_2_ content was determined by measuring absorbance at a wavelength of 390 nm using a standard curve with known concentrations.

### 2.8. RNA Sequencing

The V1 variety was selected as the subject for RNA sequencing (RNA-seq) due to its heightened sensitivity to SLs priming. RNA-seq library construction and subsequent analysis were conducted at Biomarker Technologies (Beijing, China). Briefly, RNA aliquots that exhibited concentration > 20 ng/µL, total content > 1 µg, total volume > 50 µL were used for the construction and sequencing of cDNA libraries. Both library construction and subsequent analysis were conducted at Biomarker Technologies (Beijing, China) operating on an Illumina NovaSeq6000 System (Illumina, San Diego, CA, USA). Then FastQC software (https://www.bioinformatics.babraham.ac.uk/projects/fastqc/, accessed on 15 September 2022) was used to check the quality of sequence data, and Trimmomatic software (http://www.usadellab.org/cms/?page=trimmomatic, accessed on 15 September 2022) was used to remove the adapters or reads with poor quality. Clean data were mapped to the wheat genome sequence (IWGSC RefSeq v2.1) using HISAT2 (http://daehwankimlab.github.io/hisat2/, accessed on 14 September 2022). Transcripts were then assembled with StringTie (http://ccb.jhu.edu/software/stringtie/, accessed on 15 September 2022). The calculation method for FPKM was as follows: FPKM = cDNA fragments/Mapped Reads (Millions) × Transcript Length (Kb). Differentially expressed genes (DEGs) were identified based on the following criteria: a false discovery rate (FDR) < 0.01 and fold change >2. The DEGs underwent Gene Ontology (GO) and Kyoto Encyclopedia of Genes and Genomes (KEGG) analyses using KOBAS software (v2.1.1). Furthermore, principal component analysis (PCA), pearson correlation coefficient (PCC), K-means cluster analysis, and weighted gene co-expression network analysis (WGCNA) were performed on the BMKCloud platform (https://international.biocloud.net, accessed on 4 May 2023).

### 2.9. Verification of Gene Expression by qRT-PCR

The RNA extraction was performed using TRIzol reagent (Invitrogen, Waltham, USA). For cDNA synthesis and quantitative real-time PCR (qRT-PCR), a previously published method [25] was employed. The internal reference gene utilized in this study was *ACT7*. Primer design (Table S1) was conducted using the NCBI primer design tool (http://www.ncbi.nlm.nih.gov/tools/primer-blast/, accessed on 7 July 2023).

### 2.10. Statistical Analysis

The statistical analysis was performed using the SPSS 24.0 software package (IBM SPSS Inc., Chicago, IL, USA) with Duncan’s test (*p* < 0.05).

## 3. Results

### 3.1. SLs Improving Wheat Phenotypes and Leaf Water Relations under Drought Stress

**Figure 1:**
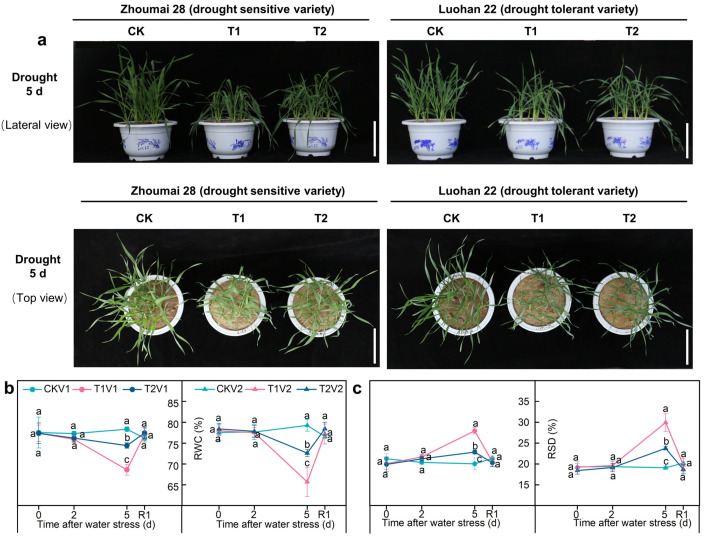
Phenotype, leaf relative water content and leaf relative saturation deficit in different treatments plants. (**a**) the photos showed the water deficit phenotype of Zhoumai 28 and Luohan 22 under normal conditions (CK), drought stress in soil (T1), and drought stress in soil with SLs priming (T2) after 5 d of drought stress; the scale bar indicates 10 cm; (**b**) leaf relative water content in different treatments; (**c**) leaf relative saturation deficit in different treatments. V1 represents Zhoumai 28, and V2 represents Luohan 22. R1 indicates 1 d after rehydration treatment. Different letters indicate significant difference at *p* < 0.05 according to one-way ANOVA followed by Duncan’s test. Data indicate mean ± SD (*n* = 3).

The two varieties showed the same response pattern to SLs application under drought stress (Figure 1a). Following 5 days of drought stress, both T1 and T2 exhibited symptoms such as loss of turgor, drooping, wilting, and darkening. Interestingly, T2 plants displayed less drought phenotypic damage compared to T1 plants. Notably, V2 demonstrated superior drought tolerance phenotype compared to V1 under identical treatment conditions (Figure 1a). The relative water content (RWC) remained relatively constant in the control group throughout the experiments. In the varieties subjected to 2 and 5 days of drought stress, the RWC followed this sequence: CK > T2 > T1 (Figure 1b). However, an inverse trend was observed with respect to relative stomatal density (RSD); after 2 and 5 days of drought stress, the sequence for the varieties was CK < T2 < T1 (Figure 1c). Compared with CK and T1, at 0 d after drought stress, T2 significantly reduced shoot dry weight and root dry weight; however, at 5 d after drought stress, T2 significantly increased shoot dry weight and root dry weight compared to T1 (Figure S3a,b). At 5 d after drought stress, T2 also significantly increased root length and root volume compared to Tl (Figure S3c,d).

### 3.2. SLs Increasing Chlorophyll Content and Photosynthesis Rate

**Figure 2:**
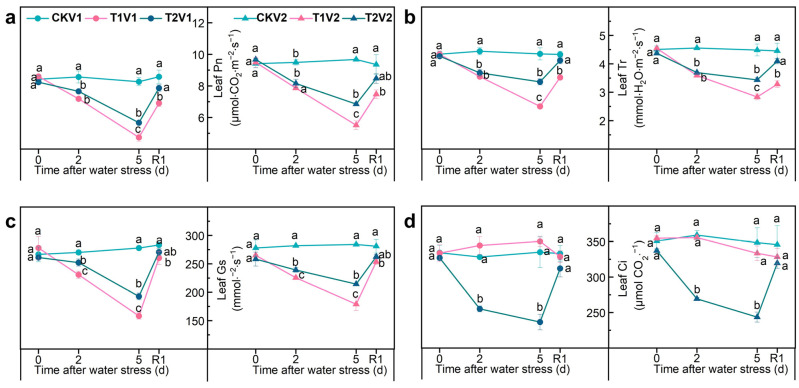
Photosynthetic performance in different treatments plants. (**a**) net photosynthetic rate; (**b**) transpiration rate; (**c**) stomatal conductance; (**d**) intercellular CO_2_ concentration. V1 represents Zhoumai 28, and V2 represents Luohan 22. R1 indicates 1 d after rehydration treatment. Different letters indicate significant difference at *p* < 0.05 according to one-way ANOVA followed by Duncan’s test. Data indicate mean ± SD (*n* = 3).

The chlorophyll a content (Figure S4a), chlorophyll b content (Figure S4b), total chlorophyll content (Figure S4c), SPAD value (Figure S4d), Pn (Figure 2a), Tr (Figure 2b), and Gs (Figure 2c) exhibited similar trends throughout the experiment (Figure S4, Figure 2); for example, the Pn of the control remained relatively stable during the entire period. However, under drought stress in both varieties, the Pn of T1 and T2 treatments decreased initially and then increased after rehydration (Figure 2a). Compared with CKV1, Pn of T1V1 and T2V1 was significantly reduced by 42.7% and 31.4%, respectively, at 5 d after drought stress. Additionally, T2V1 showed a significantly higher Pn compared to T1V1 at 5 d after drought stress (Figure 2a). Surprisingly, T2 exhibited significantly lower Ci compared with both CK and T1 in both varieties at 2 and 5 d after drought stress (Figure 2d).

### 3.3. SLs Improving Antioxidant Capacity to Scavenge ROS under Drought Stress

**Figure 3:**
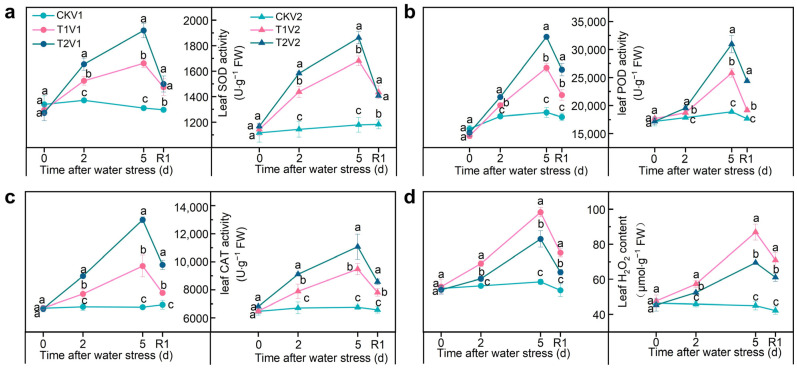
Antioxidant enzyme activity and H_2_O_2_ content in leaves under different treatments. (**a**) SOD activity; (**b**) POD activity; (**c**) CAT activity; (**d**) H_2_O_2_ content. V1 represents Zhoumai 28, and V2 represents Luohan 22. R1 indicates 1 d after rehydration treatment. Different letters indicate significant difference at *p* < 0.05 according to one-way ANOVA followed by Duncan’s test. Data indicate mean ± SD (*n* = 3).

The activities of SOD, POD, and CAT in T1 and T2 treatments remained higher under drought conditions compared with control in both varieties. However, these activities decreased after rehydration (Figure 3); for instance, the order of SOD activity at 2 and 5 under drought stress was as follows: T2 > T1 > CK. Notably, when compared with T1V1, T2V1 exhibited a significant increase in SOD activity by 8.5% and 15.6% at 2 and 5 d after drought stress, respectively. Moreover, MDA and H_2_O_2_ content typically increased with stress but decreased upon rehydration (Figure 3d, Figure S5). The H_2_O_2_ content of T2V1 showed a significant reduction by 14.4% and 18.5% at 2 and 5 d of drought stress, respectively, when compared with that of T1V1.

### 3.4. Transcriptional Regulation of SLs-Mediated Drought Relief in Wheat Leaves

A total of 2.321 billion unique reads were identified out of a total of 10.936 billion reads after sequencing, with a total map ratio ranging from 93.5% to 95.1%, and a unique map ratio ranging from 82.3% to 88.5% (Table S2). Strong reproducibility and consistency among the three biological replicates were indicated by PCC and PCA (Figure S6). More downregulated genes were observed in CK-C_VS_ T1-C after 5 d of drought stress, while more upregulated genes were observed in CK-D_VS_ T1-D after rehydration (Figure S7a). The analysis also revealed that there were 954 common DEGs between CK and T1 across three sampling points (Figure S7b), among which 209 DEGs were commonly expressed between CK and T2 at 0, 2, 5, and 6 d under water stress (Figure S7c). Additionally, there were 62 DEGs between the aforementioned 209 and 954 DEGs (Figure S7d, Table S3).

**Figure 4:**
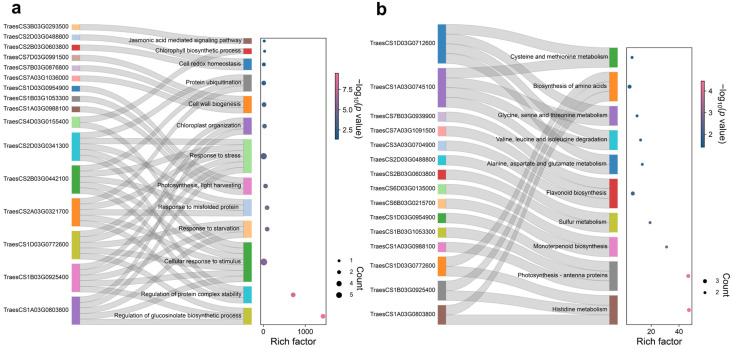
GO and KEGG enrichment of 62 DEGs. (**a**) sankey dot plot of GO analysis based on biological process; (**b**) sankey dot plot of KEGG analysis. The dot size was based on the gene count enriched in the pathway, and the color of the dot showed the pathway enrichment significance. All enrichment results were selected with the significance threshold “*p* < 0.05”.

The GO analysis revealed that the 62 DEGs were primarily enriched in biological processes related to “response to stress”, “photosynthesis, light harvesting”, “response to misfolded protein”, and “cell redox homeostasis” (Figure 4a). Additionally, the DEGs showed enrichment in molecular functions associated with “ubiquitin protein ligase activity” and “chlorophyll binding” (Figure S8a), as well as cellular components such as “photosystem I”, “photosystem II”, and “chloroplast thylakoid membrane” (Figure S8b). In KEGG analysis, the 62 DEGs were mainly enriched in “photosynthesis—antenna proteins”, and “biosynthesis of amino acids” (Figure 4b).

### 3.5. K-Means Cluster Identifying the Gene Clusters Associated with Drought Tolerance

K-means cluster analysis was performed on all differentially expressed genes (DEGs) to classify them into 15 clusters based on their expression profiles (Figure S9). This study focused specifically on cluster 2, 6, 11, and 13 due to the similarity between the expression trend of cluster 2 and 6 in terms of antioxidant enzyme activities trend, as well as the similarity between the expression trend of cluster 11 and 13 in terms of RWC trend. GO analysis revealed that cluster 2 was enriched with terms related to “response to water”, “positive regulation of response to oxidative stress”, and “regulation of photosynthesis, light reaction”. Cluster 6 showed enrichment for terms such as “response to water deprivation”, “protein stabilization”, and “photoprotection”. Cluster 11 exhibited enrichment for terms like “photosynthesis”, “response to oxidative stress”. Lastly, cluster 13 displayed enrichment for terms including “photorespiration”, and “photosynthesis, light reaction”, “regulation of removal of superoxide radicals”, and “photosynthesis, light harvesting” (Figure S10). In KEGG analysis, “peroxisome”, “carbon fixation in photosynthetic organisms”, and “glutathione metabolism” were enriched in cluster 2, “MAPK signaling pathway—plant”, and “alanine, aspartate and glutamate metabolism” were enriched in cluster 6, “porphyrin and chlorophyll metabolism”, and “carbon fixation in photosynthetic organisms” were enriched in cluster 11, and “photosynthesis”, and “porphyrin and chlorophyll metabolism” were enriched in cluster 13 (Figure S11).

### 3.6. Identification of WGCNA Modules Associated with Drought Tolerance in Wheat Leaves

**Figure 5:**
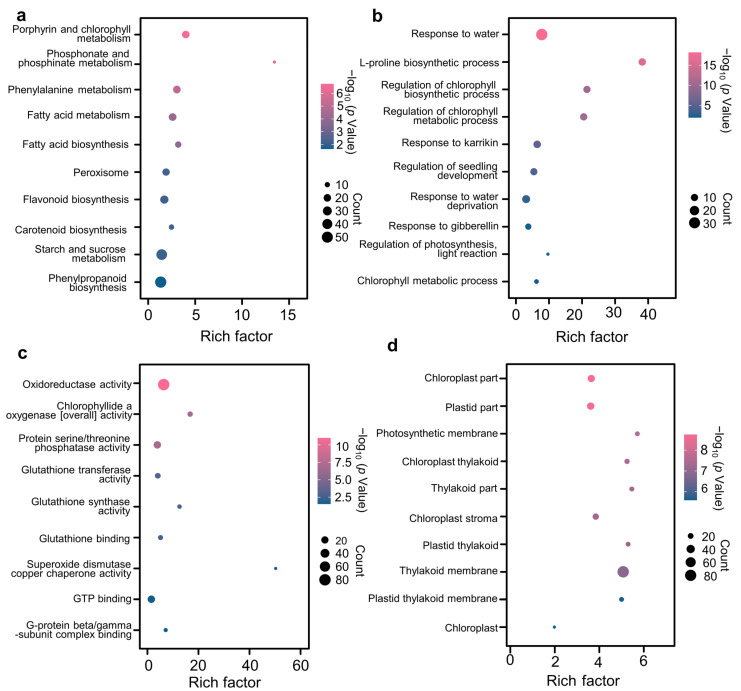
KEGG and GO enrichment of blue module by WGCNA. (**a**) KEGG enrichment analysis of blue module; (**b**) GO enrichment based on biological process of blue module; (**c**) GO enrichment based on molecular function of blue module; (**d**) GO enrichment based on cellular component of blue module. The dot size was based on the gene count enriched in the pathway, and the color of the dot showed the pathway enrichment significance. All enrichment results were selected with the significance threshold “*p* < 0.05”.

The key genes involved in SLs alleviating drought stress were also investigated through WGCNA analysis. A scale-free topological model was established with a soft threshold of 21 (Figure S12a). By employing the dynamic hierarchical tree cutting algorithm, 21 color modules were identified (Figure S12b). Among these modules, the turquoise module exhibited the highest gene count with 4433 genes, while the pale turquoise module had the lowest gene count with only 33 genes (Figure S12c). Module-trait relationships analysis revealed that the blue module displayed a significant negative correlation with RWC and SPAD value, and a significant positive correlation with SOD activity, POD activity, and RSD (Figure S12d). Next, we focused on investigating the blue module using GO and KEGG enrichment analyses. The results showed significant enrichment for KEGG terms related to “porphyrin and chlorophyll metabolism” and “peroxisome”. Additionally, there was significant enrichment for GO-biological process terms such as “response to water deprivation” and “regulation of photosynthesis, light reaction”. Furthermore, GO-molecular function terms include “glutathione synthase activity” and “superoxide dismutase copper chaperone activity”. Lastly, GO-cellular component terms like “photosynthetic membrane” and “chloroplast thylakoid” also exhibited significant enrichment (Figure 5).

A co-expression network was constructed to identify key genes involved in SLs-mediated drought stress alleviation. The top 10 hub genes with superior MCC scores were identified, including protein C2-domain ABA-related 8 and synaptotagmin-5, involved in Ca^2+^ signaling, cell number regulator 13 and senescence regulator, involved in growth and development regulation, as well as carboxyl-terminal-processing peptidase, involved in photosynthetic water oxidation (Table S4, Figure S13a). These hub genes were upregulated by drought treatments but downregulated by SLs priming under drought conditions (Figure S13b).

### 3.7. SLs Upregulating Expression of Antioxidant-Related Genes

**Figure 6:**
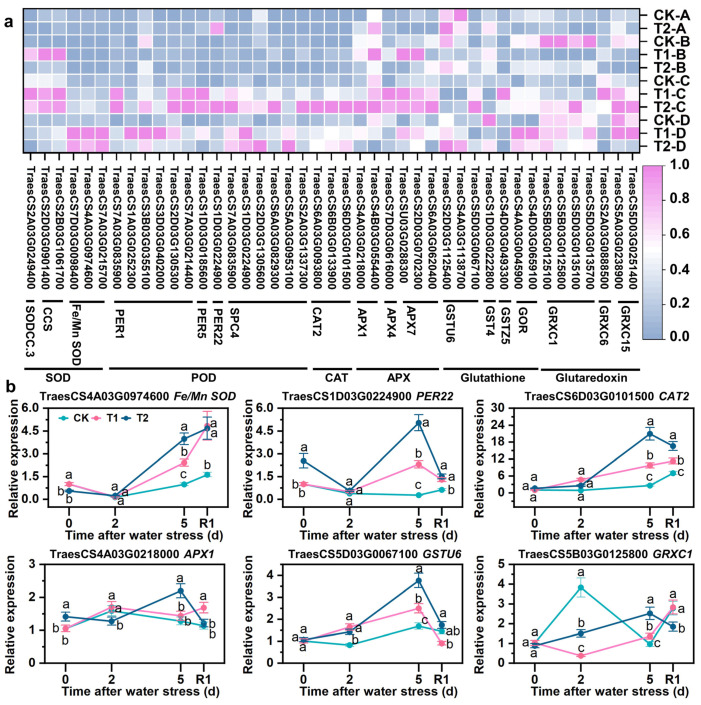
Expression differences of DEGs related to antioxidants in blue module based on WGCNA. (**a**) heatmap of the expression patterns of DEGs. FPKM values were normalized by Z-score. (**b**) qRT-PCR validation of key antioxidants genes. A, B, C, and D in coordinate maenad different sampling times, A = after water stress 0 d, B = after water stress 2 d, C = after water stress 5 d, D = rehydration for 1 d. R1 indicates 1 d after rehydration treatment. Different letters indicate significant difference at *p* < 0.05 according to one-way ANOVA followed by Duncan’s test.

This study focused on the DEGs in the blue module, as these DEGs play a crucial role in alleviating drought stress through SLs. Through GO and KEGG analysis of the blue module, numerous antioxidant-related terms were found to be enriched, and a total of 43 DEGs associated with antioxidants were identified. Under drought stress conditions, *Fe/Mn-SOD*, *PER1*, *PER22*, *SPC4*, *CAT2*, *APX1*, *APX7*, *GSTU6*, *GST4*, *GOR*, *GRXC1*, and *GRXC15* showed upregulation, which was further enhanced by SLs priming (Figure 6a). Conversely, few genes, including *SODCC.3*, *CCS*, *PER5*, *APX4*, *GSTZ5*, and *GRXC6*, were found upregulated under drought stress, but downregulated under drought stress with SLs priming (Figure 6a). The qPCR results confirmed that the expression patterns of *Fe/Mn-SOD*, *PER22*, *CAT2*, *APX1*, *GSTU6*, and *GRXC1* were consistent with those obtained from RNA-Seq data, suggesting the reliability of RNA-Seq data (Figure 6b).

### 3.8. SLs Improved Expression of Chlorophyll- and Photosynthesis-Related Genes

**Figure 7:**
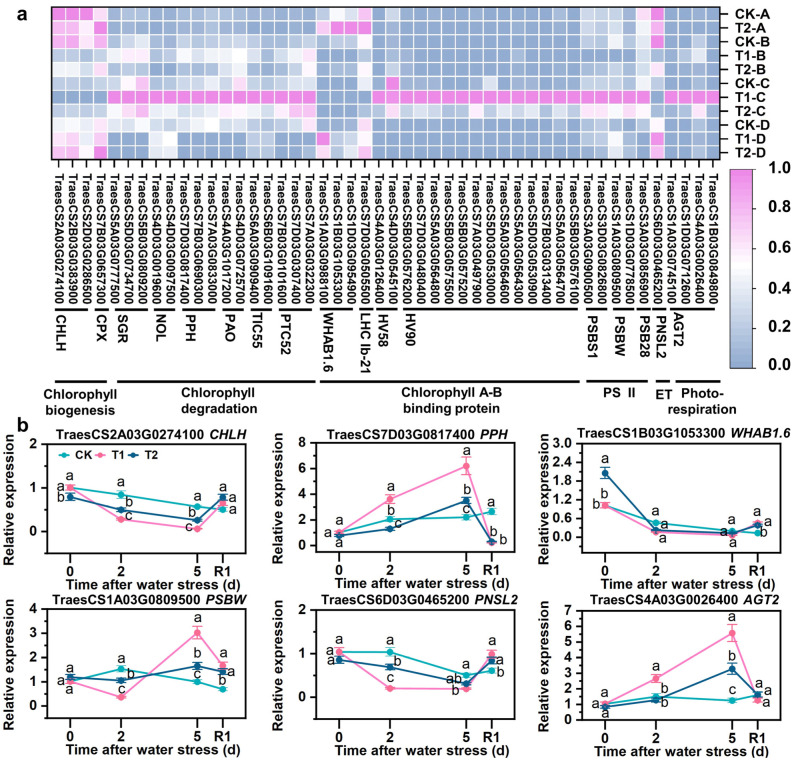
Expression differences of DEGs related to chlorophyll metabolism and photosynthesis in blue module based on WGCNA. (**a**) heatmap of the expression patterns of DEGs. FPKM values were normalized by Z-score. (**b**) qRT-PCR validation of key chlorophyll metabolism and photosynthesis genes. A, B, C, and D in coordinate maenad different sampling times, A = after water stress 0 d, B = after water stress 2 d, C = after water stress 5 d, D = rehydration for 1 d. R1 indicates 1 d after rehydration treatment. Different letters indicate significant difference at *p* < 0.05 according to one-way ANOVA followed by Duncan’s test.

The blue module of WGCNA identified 48 DEGs specifically associated with chlorophyll metabolism and photosynthesis. Under drought stress, genes involved in chlorophyll biogenesis (*CHLH*, and *CPX*), light-harvesting chlorophyll A-B binding protein-related genes (*WHAB1.6*, and *LHC Ib-21*), and electron transfer-related gene (*PNSL2*) were downregulated, but upregulated under drought with SLs priming (Figure 7a). Conversely, genes related to chlorophyll degradation (*SGR*, *NOL*, *PPH*, *PAO*, *TIC55*, and *PTC52*), thylakoids-protecting chlorophyll A-B binding protein-related genes (HV58, and HV90), light-protecting photosystem II reaction center-related genes (*PSBS1*, *PSBW*, and *PSB28*), and photorespiration-related gene (*AGT2*) were upregulated under drought stress but downregulated under drought with SLs priming (Figure 7a). The relative expression patterns of *CHLH*, *PPH*, *WHAB1.6*, *PSBW*, and *AGT2* were consistent with the FPKM values obtained from RNA-Seq results (Figure 7b).

### 3.9. SLs Upregulating Expression of Repair Protein Misfolding-Related Genes

**Figure 8:**
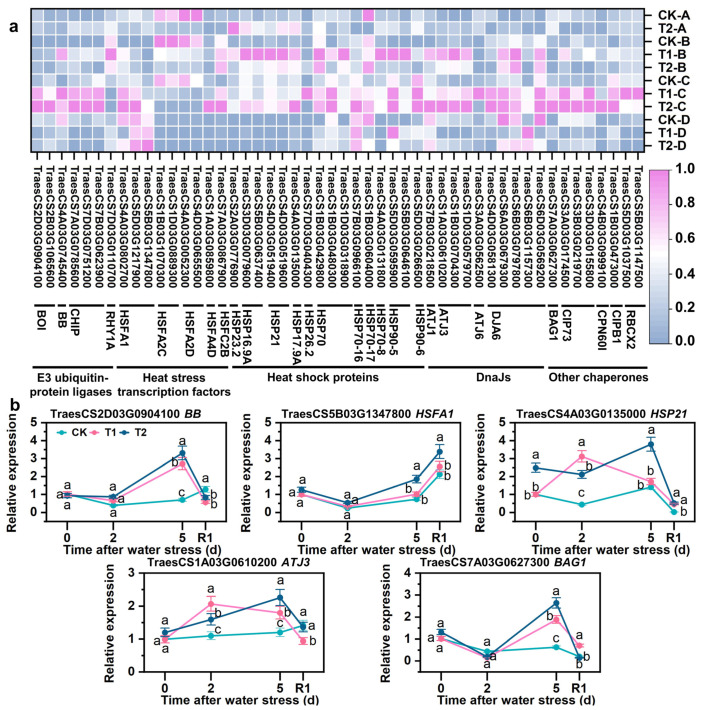
Expression differences of DEGs related to repair protein misfolding in blue module based on WGCNA. (**a**) heatmap of the expression patterns of DEGs. FPKM values were normalized by Z-score. (**b**) qRT-PCR validation of key repair protein misfolding genes. A, B, C, and D in coordinate maenad different sampling times, A = after water stress 0 d, B = after water stress 2 d, C = after water stress 5 d, D = rehydration for 1 d. R1 indicates 1 d after rehydration treatment. Different letters indicate significant difference at *p* < 0.05 according to one-way ANOVA followed by Duncan’s test.

The blue module comprises 50 DEGs involved in the repair of protein misfolding. Under drought stress, the upregulation of E3 ubiquitin-protein ligase coding genes (*BB*, *CHIP*, and *RHY1A*), heat stress transcription factors-coded genes (*HSFA1*, *HSFA4D*, and *HSFC2B*), heat shock proteins-coded genes (*HSP23.2*, *HSP16.9A*, *HSP17.9A*, *HSP21*, *HSP70*, *HSP70-16*, *HSP70-17*, *HSP70-8*, *HSP90-5*, and *HSP90-6*), DnaJ-coded genes (*ATJ1*, *ATJ3*, and *DJA6*), other chaperones (*BAG1*, *CIP73*, *CIPB1*, and *CPN60I*), and SLs priming further enhanced the expression of these genes under drought stress conditions (Figure 8a). The qRT-PCR results confirmed that the expression trends of *BB*, *HSFA1*, *HSP21*, *ATJ3*, and *BAG1* were consistent with those obtained from RNA-seq analysis; specifically, drought stress upregulated their expression levels, which were further increased by SLs priming (Figure 8b).

## 4. Discussion

### 4.1. SLs Improved Scavenging Capacity of ROS Responding to Drought Stress

Under drought stress, plants can accumulate a substantial amount of ROS, leading to cellular oxidative damage [26,27]. Antioxidants such as SOD [24], POD [5], CAT [25], glutathione [28], and glutaredoxin [29] play a crucial role in scavenging ROS and alleviating drought-induced damage. The current study demonstrated that under drought conditions, SLs priming significantly enhanced the activities of SOD, POD, and CAT, while concurrently reducing the levels of H_2_O_2_ and MDA under drought environment (Figure 3). These results are consistent with previous studies in wheat, which suggested that SLs improve antioxidant enzyme activity while reducing ROS production [14,19,20,21]. This study revealed enrichment of terms such as “Cell redox homeostasis”, “positive regulation of response to oxidative stress”, “response to oxidative stress”, “regulation of removal of superoxide radicals”, “peroxisome”, “glutathione metabolism”, and “glutathione synthase activity” (Figure 4 and Figure 5, Figures S8–S11). Similarly, in soybean subjected to alkaline stress with SLs application, the enrichment analysis indicated upregulation of genes associated with “peroxidase activity”, “response to oxidative stress”, and “hydrogen peroxide metabolic pathway” [18]. Furthermore, a series of antioxidants-coded genes (*Fe/Mn-SOD, PER1*, *PER22*, *SPC4*, *CAT2*, *APX1*, *APX7*, *GSTU6*, *GST4*, *GOR*, *GRXC1*, and *GRXC15*) were upregulated under drought stress with SLs priming (Figure 7). These findings are similar with previous studies suggesting that SLs induce upregulation of antioxidant-related genes such as *SOD*, *POD*, *CAT*, *APX*, and *GRX2* under salt stress [30].

### 4.2. SLs Restrained Degradation of Chlorophylls and Increased Biosynthesis

Chlorophyll serves as the fundamental basis for photosynthesis, and alterations in chlorophyll content play a pivotal role in evaluating plant growth and drought tolerance. Previous studies have demonstrated that SLs enhance chlorophyll content under drought stress in various crops, including wheat [21], maize [31], rapeseed [32], grape [33], and alfalfa [34]. In this study, we observed a decrease in chlorophyll content (chlorophyll a, chlorophyll b, total chlorophyll, and SPAD value) under drought conditions; however, priming with SLs significantly increased these indices under drought stress (Figure S4). Enrichment analysis revealed the involvement of key processes such as “Porphyrin and chlorophyll metabolism”, “regulation of chlorophyll biosynthetic process”, “regulation of tetrapyrrole biosynthetic process”, and “protoporphyrinogen IX biosynthetic process” (Figure 4 and Figure 5, Figures S8–S11). Furthermore, SLs treatment downregulated genes associated with chlorophyll degradation (*SGR*, *NOL*, *PPH*, *PAO*, *TIC55*, and *PTC52*), while upregulating genes involved in chlorophyll biogenesis (*CHLH*, and *CPX*) in the leaves (Figure 8). These findings suggested that SLs can enhance chlorophyll content by upregulating genes related to its biogenesis while downregulating those associated with its degradation under drought stress.

### 4.3. SLs Promoted Photosynthesis by Increasing Electron Transport and Inhibiting Photorespiration

Drought stress induces the generation of ROS, resulting in impaired photosystem II (PSII) and subsequent reduction in wheat’s photosynthetic capacity [35,36]. The improvement of leaf water relations could increase Pn in wheat under drought stress [37]. Interestingly, application of SLs has been demonstrated to enhance photosynthetic performance under drought conditions [19,32,34,38,39]. In this study, SLs priming could increase antioxidant activity to scavenge excess ROS, improve leaf water relations, and enhance chlorophyll content under drought stress; these changes led to the improvement of photosynthetic performance (Pn, Tr, and Gs), and then increased root and shoot biomass (Figure 1, Figure 2 and Figure 3, Figures S3 and S4). Notably, no significant difference was observed in Ci between subjects treated with drought alone and control; however, SLs priming resulted in a significant decrease in Ci under stress conditions. These findings suggested that the decrease in photosynthetic efficiency caused by drought is primarily attributed to stomatal limitation, whereas stomatal limitation is no longer a factor in the decrease of photosynthetic efficiency after SLs application, which partly explains that SLs can improve photosynthetic performance [40]. Furthermore, DEGs involved in various additional pathways related to photosynthesis were identified, including “photosynthesis, light harvesting”, “photosystem I”, “photosystem II”, “chloroplast thylakoid membrane”, “photosynthesis—antenna proteins”, “regulation of photosynthesis, light reaction”, “photoprotection”, “photosynthesis”, “photorespiration”, and “carbon fixation in photosynthetic organisms” (Figure 4 and Figure 5, Figures S8–S11). The enrichment analysis revealed similar upregulation of pathways such as “Carbon fixation in photosynthetic organisms”, “photosynthesis”, and “photosynthesis—antenna proteins” under both drought stress with foliar application of SLs treatments reported for grapevine and alfalfa plants [33,41].

Drought and SLs exhibited a complex regulatory pattern in relation to genes associated with photosynthesis. Under drought stress, the expression of *PSBS1*, *PSBW*, and *AGT2* were upregulated; however, their expressions were suppressed when primed with SLs. The expression of *PNSL2* decreased under drought stress but increased under drought stress with SLs priming. The elevated expression levels of *PSBS* and *PSBW* under stress is a crucial mechanism for photoprotection [42,43], while alanine glyoxylate aminotransferase (*AGT*) plays a central role in photorespiration [44]. *PNSL* participates in photosynthesis by mediating PSI cyclic electron transport [45]. In summary, drought upregulated the expression of genes associated with photoprotection; however, this effect was alleviated following SLs priming and subsequent downregulation of these photoprotection genes. Moreover, despite the strong perturbation of electron transport in photosynthesis, SLs may enhance electron transport efficiency, thereby improving photosynthesis by upregulating *PNSL2* expression.

### 4.4. HSFs and Chaperones Collaborated to Maintain the Turnover of Proteins

It is widely acknowledged that newly translated proteins undergo misfolding due to excessive ROS induced by drought [26,46,47,48]. Plants have developed a crucial mechanism involving E3 ubiquitin-protein ligase, heat shock factors (HSFs), and chaperones to regulate protein folding and degradation in response to adverse conditions [49,50,51]. According to a classical model, the chaperone-associated E3 ubiquitin ligase CHIP ubiquitinates soluble misfolded proteins associated with chaperone molecules and targets them for degradation through the 26S proteasome during stress [52]. The overexpression of E3 ubiquitin ligases, HSF, HSP, and DnaJ has been demonstrated to enhance root length, chlorophyll content, and antioxidant enzyme activity in transgenic plants, but reduce the production of MDA and ROS under stress [53,54,55,56,57]. In this study, several metabolic pathways were enriched, including “response to misfolded protein”, “protein stabilization”, “misfolded protein binding”, “ubiquitin protein ligase activity”, “ubiquitin-like protein ligase activity”, “ubiquitin-like protein transferase activity”, and “ubiquitin-protein transferase activity” (Figure 4 and Figure 5, Figures S8–S11). Furthermore, SLs priming upregulated the expression of genes encoding E3 ubiquitin-protein ligases (*BB*, *CHIP*, and *RHY1A*), heat stress transcription factor-coded genes (*HSFA1*, *HSFA4D*, and *HSFC2B*), heat shock proteins-coded genes (*HSP23.2*, *HSP16.9A*, *HSP17.9A*, *HSP21*, *HSP70*, *HSP70-16*, *HSP70-17*, *HSP70-8*, *HSP90-5*, and *HSP90-6*), DnaJ-coded genes (*ATJ1*, *ATJ3*, and *DJA6*), other chaperones (*BAG1*, *CIP73*, *CIPB1*, and *CPN60I*) compared with drought stress (Figure 8). This study also found that SLs priming increased chlorophyll content, photosynthetic performance, root length, dry weight (Figure 2 and Figure S3), while reducing membrane lipid peroxidation and oxidative damage (Figure 3 and Figure S5). To sum up, this study suggested that upregulated expression of maintenance protein turnover genes contributes to alleviating drought stress by promoting photosynthesis, ROS scavenging, and biomass accumulation. To our knowledge, this study provides novel insights into how SLs application alleviates stress by regulating E3 ubiquitin-protein ligases, HSFs, and chaperones involved in maintaining proper protein turnover.

### 4.5. Physiological, Phenotypic, and Transcriptome Analysis Revealed the Comprehensive Mechanism of Sls Alleviating Drought Stress in Wheat

**Figure 9:**
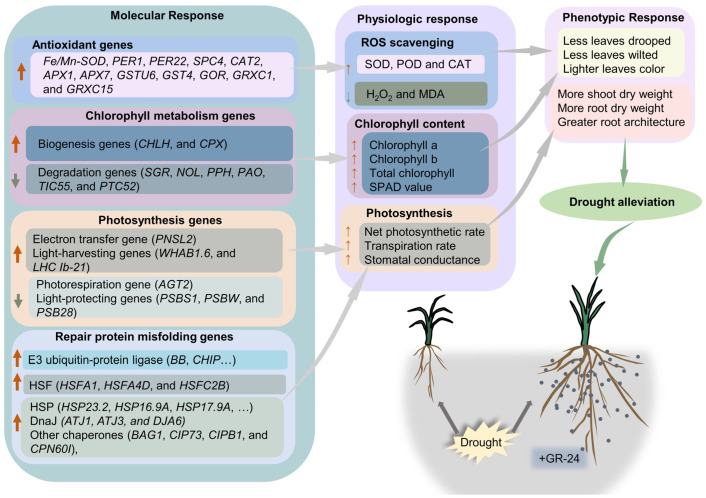
Schematic illustration of a hypothetical mechanism for SLs-mediated drought alleviation in wheat. Note: the bold orange arrows indicate upregulated genes; the bold gray arrows indicate downregulated genes; thin orange arrows indicate increase; thin gray arrows indicate decrease.

This study provides insights into the physiological and molecular mechanisms underlying the alleviation of drought stress by SLs priming in wheat (Figure 9). Specifically, SLs upregulated the expression of genes related to antioxidants (SOD-, POD-, CAT-, glutathione- and glutaredoxin-coded genes), increased the activity of SOD, POD, and CAT, and reduced H_2_O_2_ and MDA accumulation under drought stress. Furthermore, SLs enhanced the expression of genes involved in chlorophyll biogenesis and photosynthesis while suppressing the expression of genes associated with chlorophyll degradation and photorespiration. These molecular changes ultimately improved photosynthetic performance. Then the physiological and molecular changes led to a beneficial phenotypic response to drought stress.

## 5. Conclusions

In summary, SLs application effectively alleviated drought-induced damages in wheat. SLs upregulated the expression of antioxidant-related genes and repair protein misfolding-related genes; moreover, SLs improved the expression of chlorophyll- and photosynthesis-related genes. These molecular changes increased antioxidant enzymes activity, chlorophyll content, and photosynthetic rate. Eventually, the drought-damaged phenotypes of leaves and roots were alleviated. Overall, these findings provide novel insights into the beneficial effects of SLs on wheat plants subjected to drought stress; however, additional research is required for cloning and functional validation of genes associated with SLs-mediated alleviation of drought stress.

## Data Availability

Data are contained within the article.

## Supplementary Materials

The following supporting information can be downloaded at: https://www.mdpi.com/article/10.3390/antiox12101884/s1, Table S1. Primers used in this study; Table S2. Overview of RNA-seq data; Table S3. Descriptions of 62 DEGs; Table S4. Hub genes annotation; Figure S1. Changes in field capacity of potted plants; Figure S2. Illustration of the experimental design and procedure; Figure S3. Dry weight and root architecture; Figure S4. Chlorophyll content and SPAD value of wheat top expended leaves between different treatments; Figure S5. MDA content of wheat leaves between different treatments; Figure S6. Quality evaluation of RNA-seq data; Figure S7. Comparison of differentially expressed genes between CK, T1, and T2; Figure S8. GO annotation based on molecular function and cellular component of 62 DEGs; Figure S9. K-means clustering of global DEGs expression; Figure S10. GO enrichment based on biological processes of key clusters by K-means clustering; Figure S11. KEGG enrichment of key clusters by K-means clustering; Figure S12. WGCNA process; Figure S13. Hub genes based on WGCNA.
