# Supplementary material for: Physiological and RNA-Seq Analyses on Exogenous Strigolactones Alleviating Drought by Improving Antioxidation and Photosynthesis in Wheat (Triticum aestivum L.)
Source: Antioxidants (Basel). 2023 Oct 20;12(10):1884. doi: 10.3390/antiox12101884 (PMC10604895; doi:10.3390/antiox12101884)
Supplement: Supplementary file 1 [file antioxidants-12-01884-s001.zip › antioxidants-2658661-supplementary.pdf]

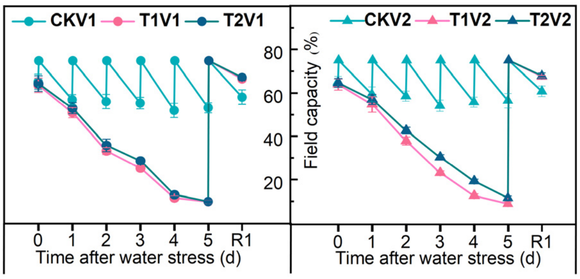

Figure S1. Changes in field capacity of potted plants. The X-axis was the time after water treatment. Data indicate mean ± SD (n = 3).

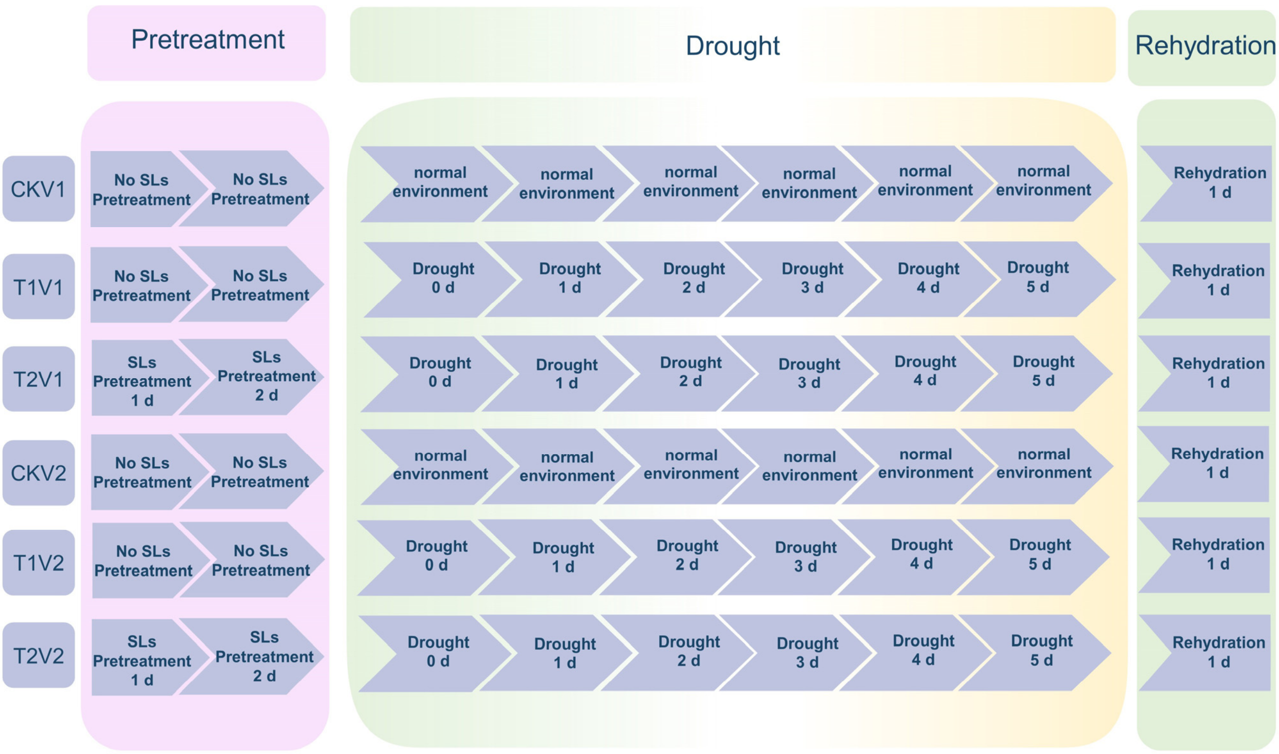

Figure S2. Illustration of the experimental design and procedure. Normal environment mean watering soil content to 75% field capacity daily.

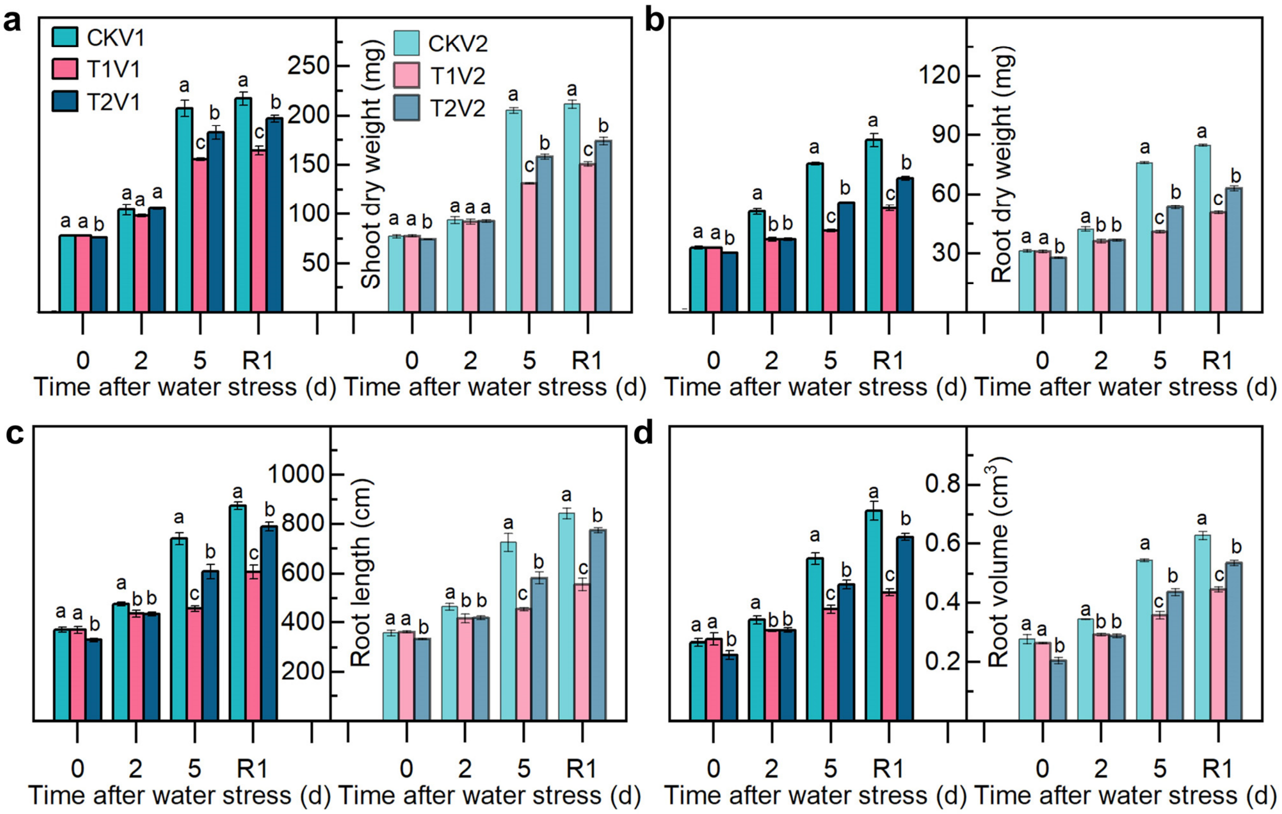

Figure S3. Dry weight and root architecture. a, shoot dry weight; b, root dry weight; c, root length; d, root volume. Different letters indicate significant difference at $p < 0.05$ according to one-way ANOVA followed by Duncan's test. Data indicate mean ± SD (n = 3).

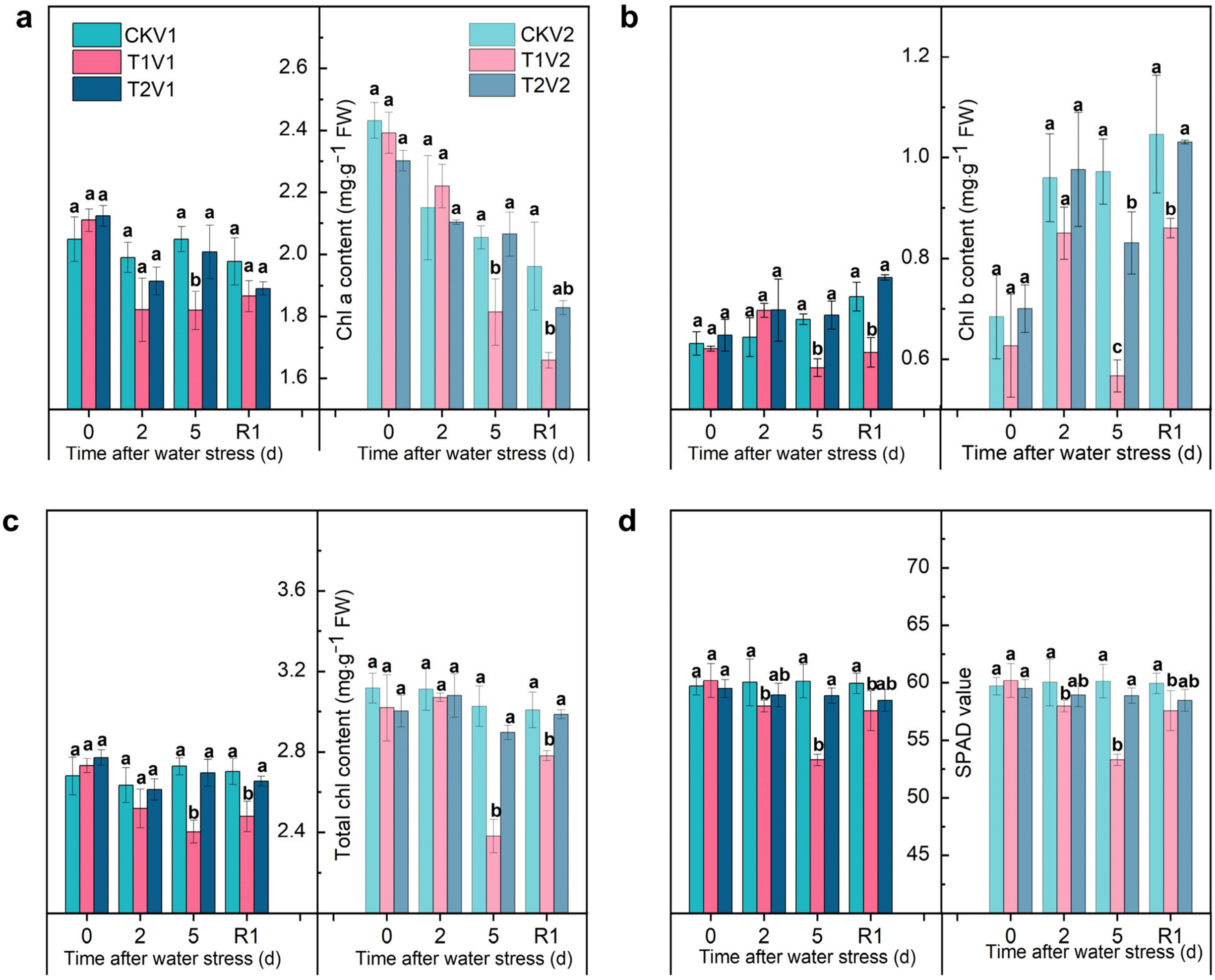

Figure S4. Chlorophyll content and SPAD value of wheat top expended leaves between different treatments. a, chlorophyll a content; b, chlorophyll b content; c, total chlorophyll content; d, SPAD value. Different letters indicate significant difference at $p < 0.05$ according to one-way ANOVA followed by Duncan's test. Data indicate mean ± SD (n = 3).

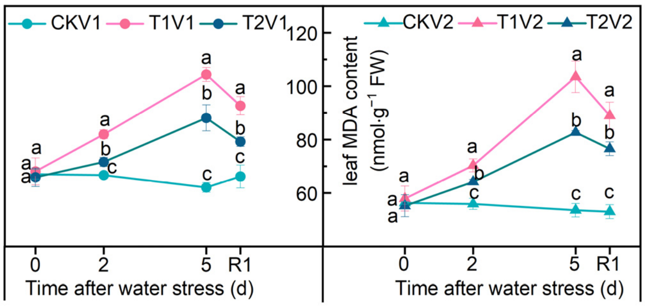

Figure S5. MDA content of wheat leaves between different treatments. Different letters indicate significant difference at $p < 0.05$ according to one-way ANOVA followed by Duncan's test. Data indicate mean ± SD (n = 3).

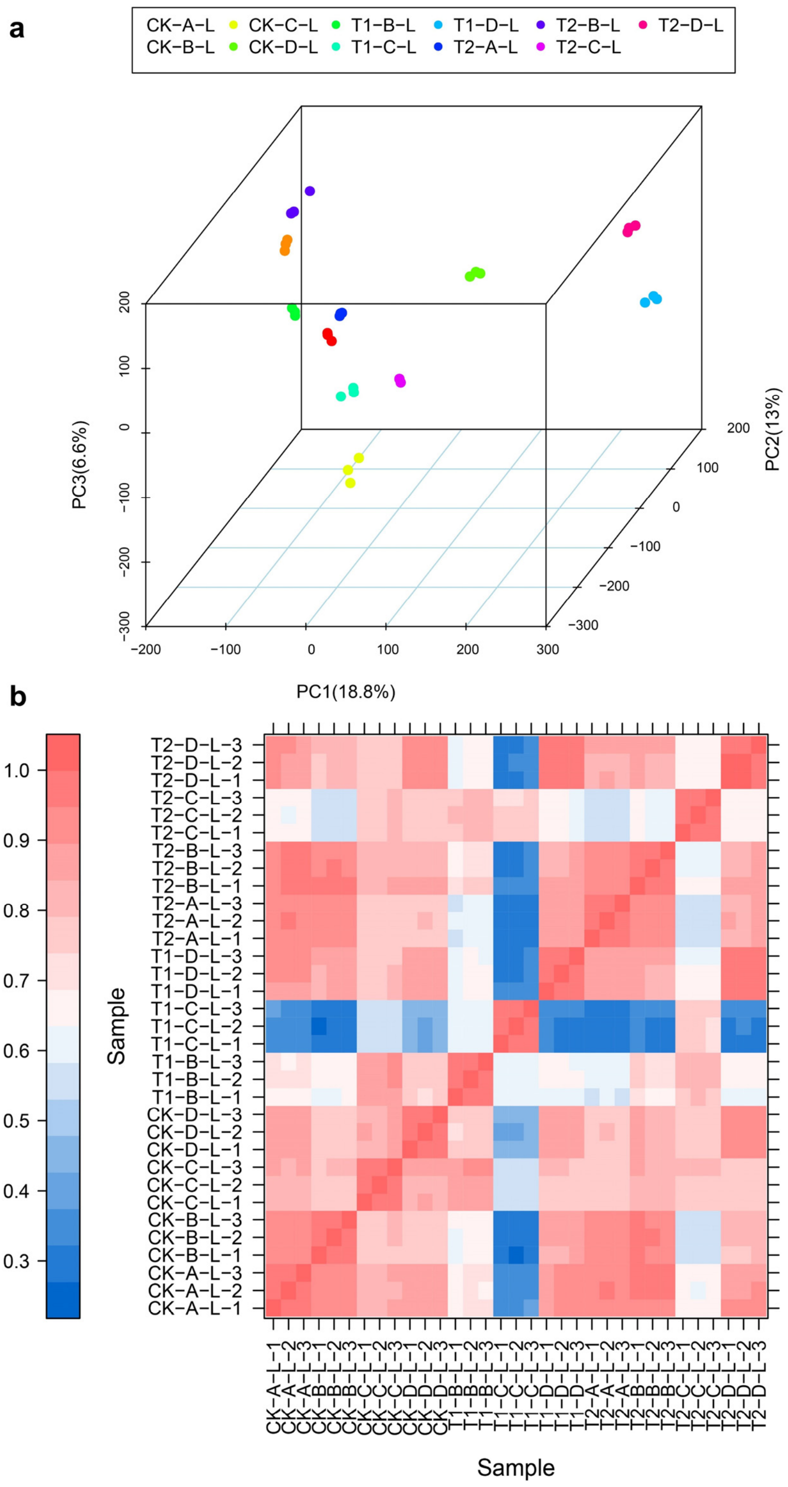

Figure S6. Quality evaluation of RNA-seq data. a, PCA displaying the major composition of variance; b, representation of correlation matrix between RNA-seq samples based on Pearson correlation coefficients. A, B, C and D in coordinate maenad different sampling times, A= after water stress 0 d, B= after water stress 2 d, C= after water stress 5 d, D= rehydration for 1 d.

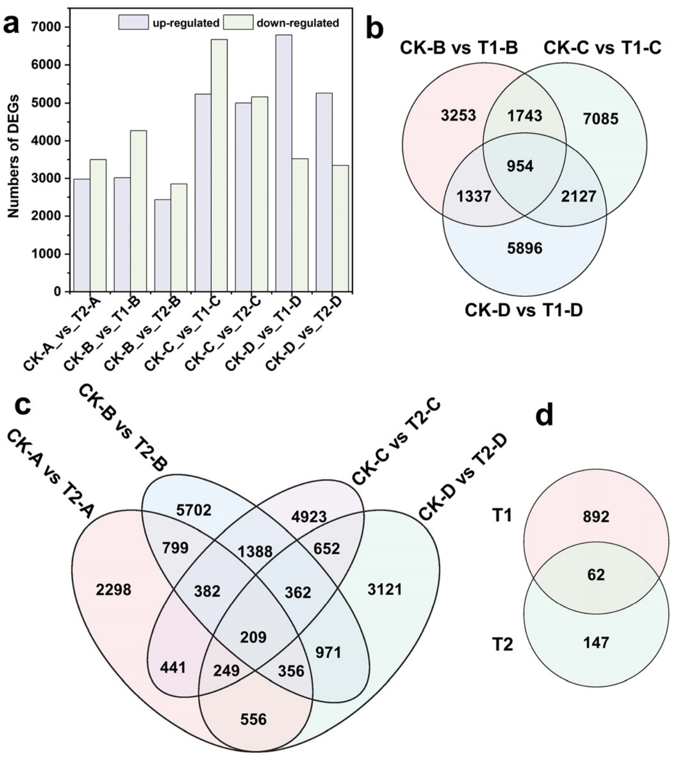

Figure S7. Comparison of differentially expressed genes between CK, T1, and T2. a, the number of up-regulated and down-regulated DEGs. b, Venn diagrams of common and unique DEGs between T1 and CK at different time; c, Venn diagrams of common and unique DEGs between T2 and CK at different time; d, Venn diagrams between T1 and T2 of above overlap DEGs. A, B, C and D in coordinate or Venn diagrams maenad different sampling times, A= after water stress 0 d, B= after water stress 2 d, C= after water stress 5 d, D= rehydration for 1 d.

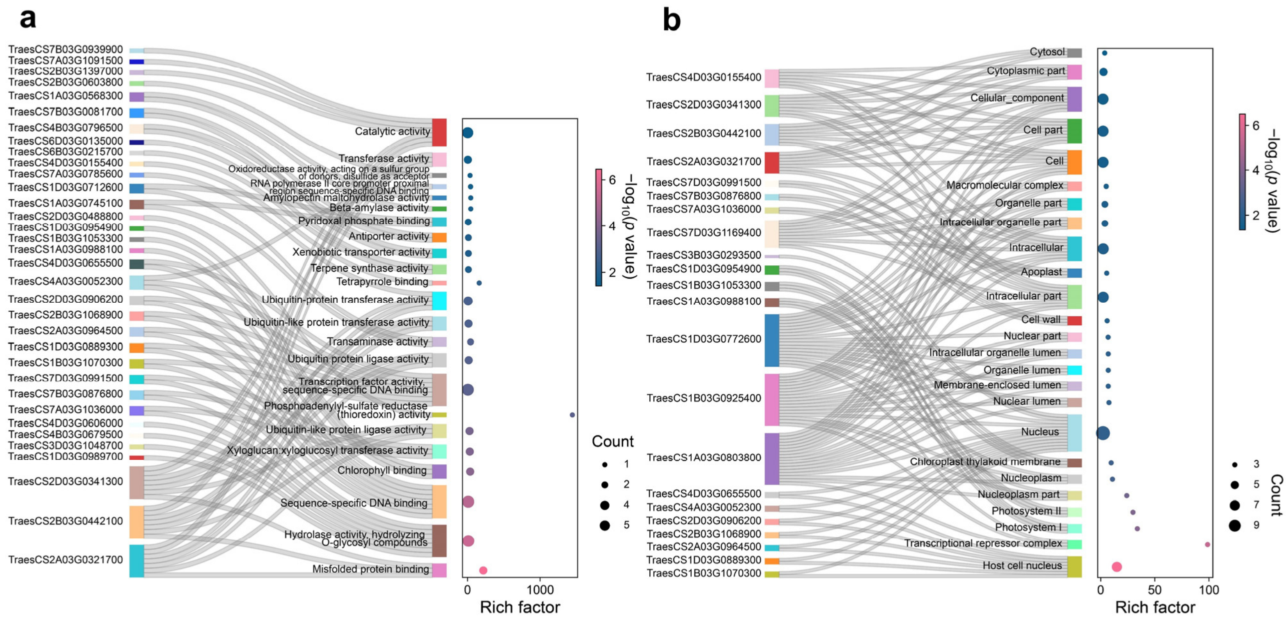

Figure S8. GO annotation based on molecular function and cellular component of 62 DEGs. a, sankey dot plot of GO analysis based on molecular function; b, sankey dot plot of GO analysis based on cellular component. The dot size is based on the gene count enriched in the pathway, and the color of the dot shows the pathway enrichment significance. All enrichment results were selected with the significance threshold "$p < 0.05$".

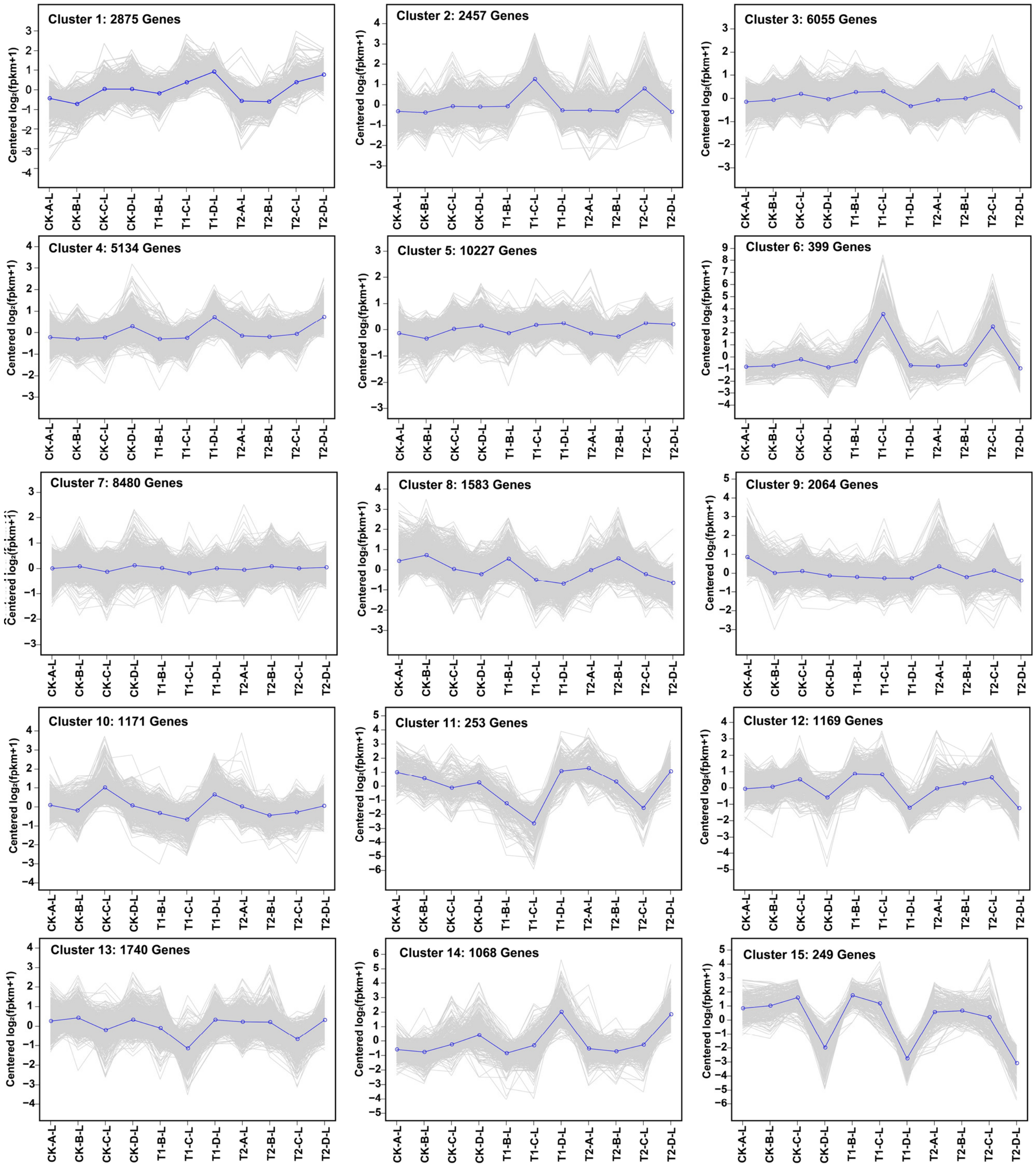

Figure S9. K-means clustering of global DEGs expression. The numbers of genes in each cluster are shown on the top of each graph.

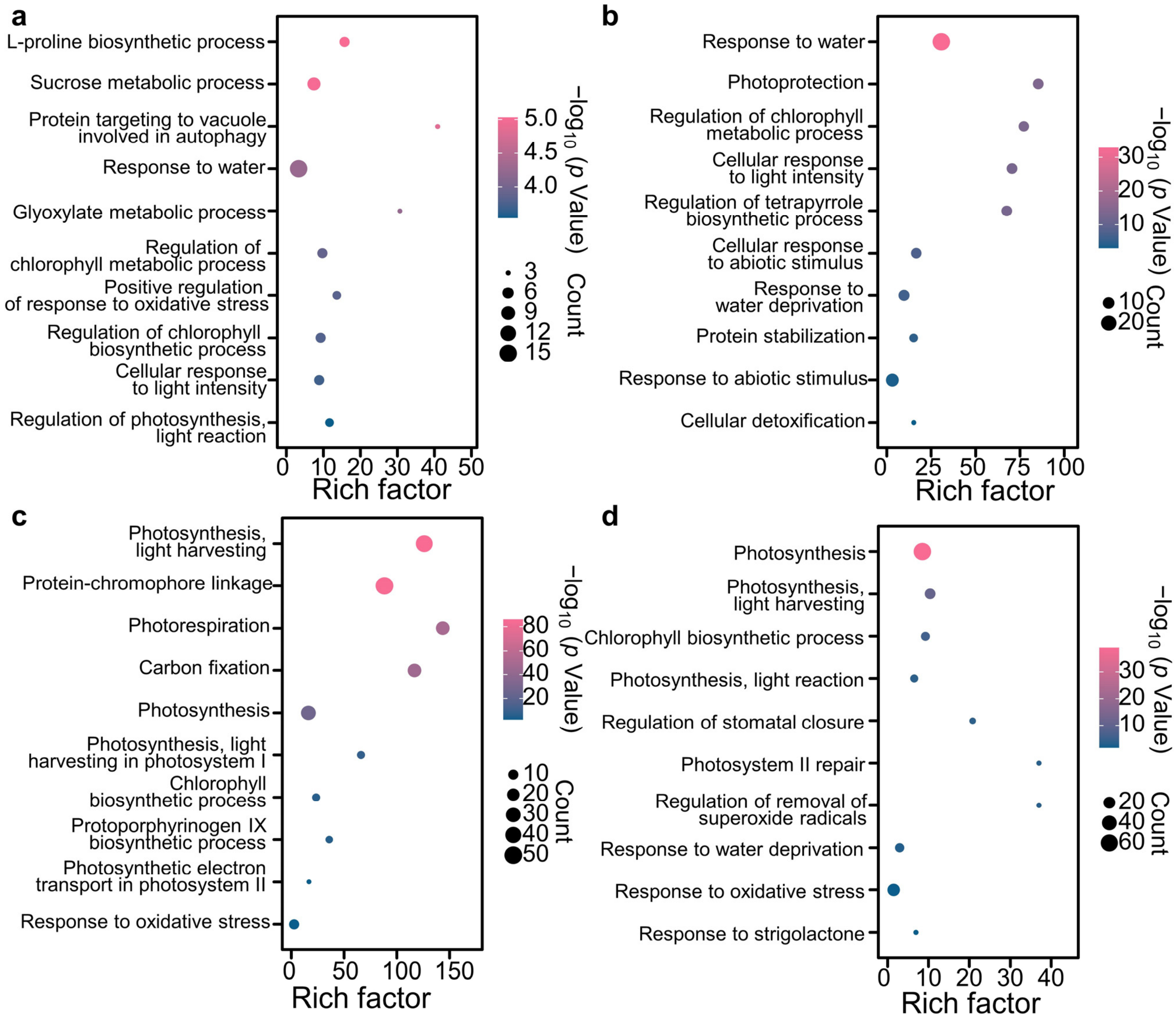

Figure S10. GO enrichment based on biological processes of key clusters by K-means clustering. a, GO enrichment analysis of cluster 2; b, GO enrichment analysis of cluster 6; c, GO enrichment analysis of cluster 11; d, GO enrichment analysis of cluster 13. The dot size is based on the gene count enriched in the pathway, and the color of the dot shows the pathway enrichment significance. All enrichment results were selected with the significance threshold "$p < 0.05$".

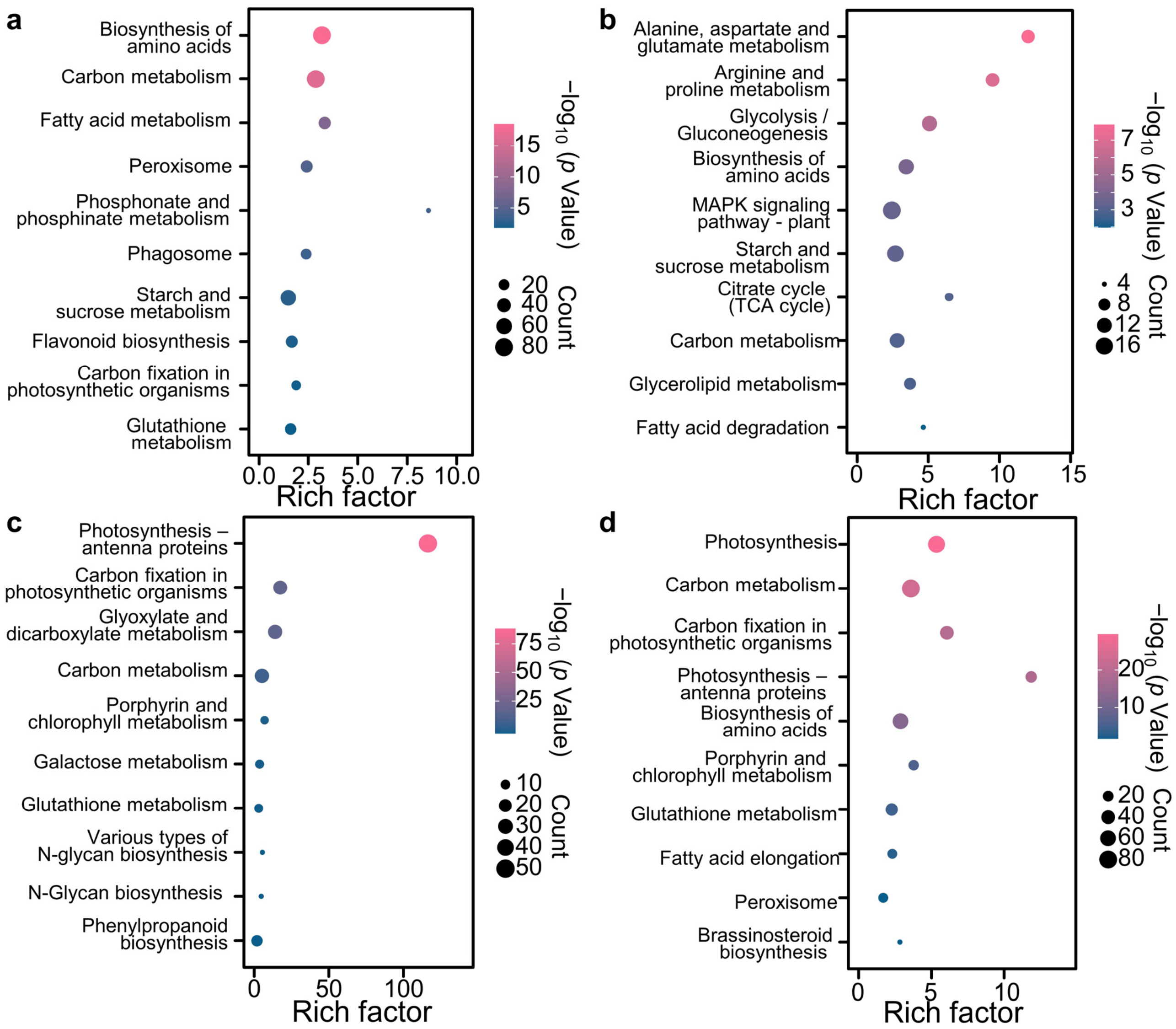

Figure S11. KEGG enrichment of key clusters by K-means clustering. a, KEGG enrichment analysis of cluster 2; b, KEGG enrichment analysis of cluster 6; c, KEGG enrichment analysis of cluster 11; d, KEGG enrichment analysis of cluster 13. The dot size is based on the gene count enriched in the pathway, and the color of the dot shows the pathway enrichment significance. All enrichment results were selected with the significance threshold "$p < 0.05$".

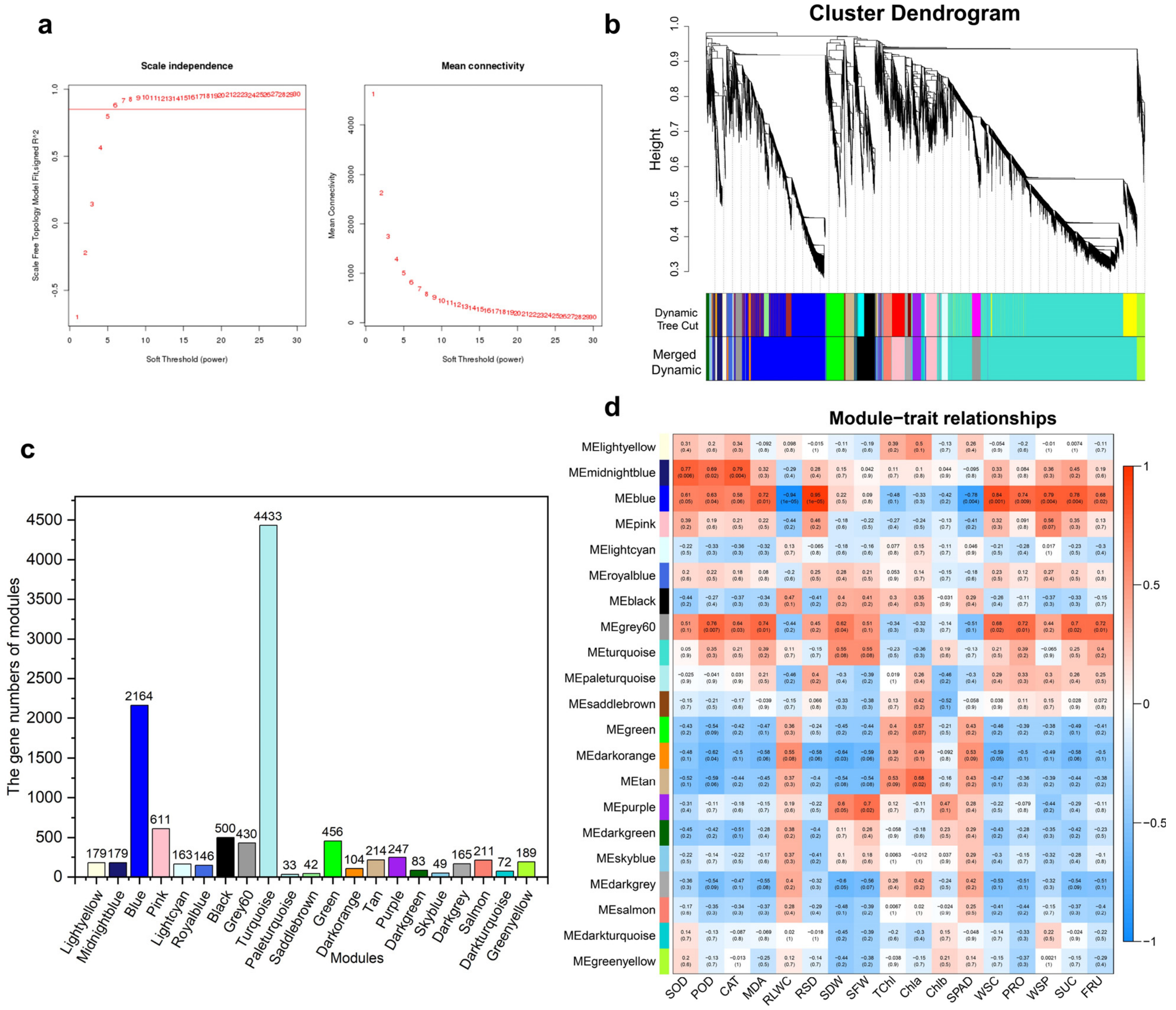

Figure S12. WGCNA process. a, network topology analysis of various soft thresholds. The left panel shows the scale-free fitting index (Y-axis) as a function of the soft threshold (X-axis). The right panel shows average connectivity (Y-axis) as a soft threshold (X-axis); b, systematic clustering tree of gene, gene network/module generated by dynamic shear method and gene network/module after merger; c, the genes number of module; d, relationships between modules and traits. The darker colors indicate higher correlation coefficients. Numbers represent Pearson's correlation coefficients R^2-values and the p-value for the correlation

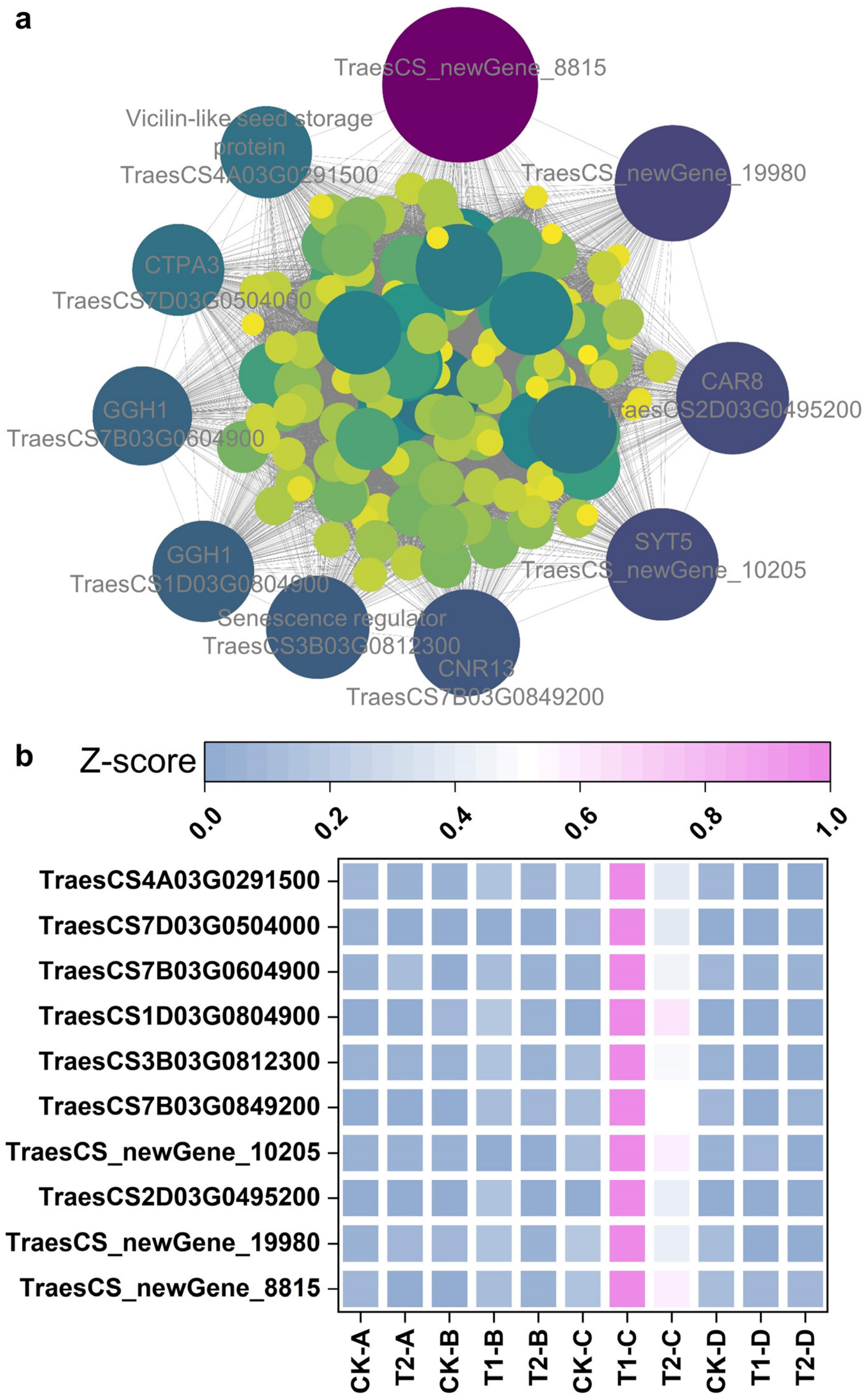

Figure S13. Hub genes based on WGCNA. a, Co-expression networks of the top 10 hub genes in the blue module, the dark carmine color and big circle area indicate a higher MMC score calculated with the CYTOHUBAA plugin of the CYTOSCAPE software. b, the heatmap of gene FPKM expression in blue module, the expression level of the same gene was normalized by Z-score. A, B, C and D in coordinate maenad different sampling times, A= after water stress 0 d, B= after water stress 2 d, C= after water stress 5 d, D= rehydration for 1 d.

Table S1. Primers used in this study.

GeneID	Gene name	Description	Forward primer	Reverse primer
TraesCS2A03G0274100	*CHLH*	Magnesium-chelatase	CTTCATCGGCTCGCTCATCT	GGAAGAAGGGGCTCTTGGAC
TraesCS7D03G0817400	*PPH*	Pheophytinase	AAGTGGCGATTCCTGGTCTG	GGTCACGCCACAAGTCTACA
TraesCS1B03G1053300	*WHAB1.6*	Chlorophyll A-B binding protein	TACTTGACCGGCGAGTTTCC	ATAGAGAACATGGCGAGGCG
TraesCS1A03G0809500	*PSBW*	Photosystem II reaction cente	TCTGGTGGGGTTTGCATTGT	TCCTCTTGGGTCCAATTGCC
TraesCS6D03G0465200	*PNSL2*	Photosynthetic NDH	GTACTGTTCTACGTGCGGCT	ACGTCATCCAGCGACTTCAG
TraesCS4A03G0026400	*AGT2*	Alanine glyoxylate aminotransferase	TTGCAGACGTGGAAGTACCC	ACAACTGCACCGAGTGGTAG
TraesCS4A03G0974600	*Fe/Mn SOD*	Superoxide dismutases	AAGGAGGCCTACAACAACGG	TGTGAACCACCTCGAGCTTC
TraesCS1D03G0224900	*PER22*	Peroxidase	CCAACTTTTTCGAAGCCGGG	GGTAGTATCGTCGAGCGTGG
TraesCS6D03G0101500	*CAT2*	Catalase	ACCCAGGACCTCTACGACTC	AGCTGCTCGTTCTCGTTGAA
TraesCS4A03G0218000	*APX1*	L-ascorbate peroxidase	GAGGTCTGGCTTTGAGGGAC	GGAAGACAGGGTCAGTCAGC
TraesCS5D03G0067100	*GSTU6*	Glutathione S-transferase	CCTCAGCTACGAGTACGTGG	AACACCCCCTCCAAGGTTTC
TraesCS5B03G0125800	*GRXC1*	Glutaredoxin	ATGGAGCAGGTGACGAAGC	GTTCCACCACCGTCGGATT
TraesCS2D03G0904100	*BB*	E3 ubiquitin-protein ligase	AGGGAAGAAGGAAGCGGTTG	TCGCGGTTCTTGTAAGCCAT
TraesCS5B03G1347800	*HSFA1*	Heat stress transcription factor	ACCATCGCATGCAAACAACC	CCTCCATCCCAAACTTCCCC
TraesCS4A03G0135000	*HSP21*	heat shock protein	TCGCGCTGGATTTGACATCT	GTCCATCGTGTCCAGCATCT
TraesCS1A03G0610200	*ATJ3*	DnaJ	CGCGTGCTCGACTTCTATCT	TTTGTTTGATTGCCCCGCAG
TraesCS7A03G0627300	*BAG1*	BAG domain	AGAAGAACGTGGAGACGCTG	GGACAGCAGGTCGAACGTAT
TraesCS1A03G0693200	*ACT7*	β-Actin (internal control gene)	TATGCCAGCGGTCGAACAAC	ATGTGGCGCTAGCTAACACTG

Table S2. Overview of RNA-seq data

Sample	Total Reads	Map reads	Q20(%)	Q30(%)	GC (%)	Total map (%)	Unique map (%)	Multi_map (%)
CK-A-L-1	71268332	33695525	97.79	94.29	58.08	94.56%	86.98%	7.58%
CK-A-L-2	73686506	34695620	97.31	92.9	57.51	94.17%	87.18%	6.99%
CK-A-L-3	72842122	34112247	97.15	92.57	57.29	93.66%	87.06%	6.60%
CK-B-L-1	69827340	32963516	97.25	92.62	56.82	94.41%	84.54%	9.87%
CK-B-L-2	65847614	31172133	97.57	93.43	56.92	94.68%	85.44%	9.24%
CK-B-L-3	70378370	33242740	97.37	92.92	57.11	94.47%	84.94%	9.53%
CK-C-L-1	83826314	39719420	97.76	93.83	56.63	94.77%	87.79%	6.98%
CK-C-L-2	70733726	33284597	97.31	92.76	56.38	94.11%	84.97%	9.14%
CK-C-L-3	71279484	33475992	97.4	92.96	56.58	93.93%	86.34%	7.59%
CK-D-L-1	67094998	31641757	97.38	92.92	56.24	94.32%	87.29%	7.03%
CK-D-L-2	69885212	32931529	97.26	92.61	56.42	94.24%	87.19%	7.06%
CK-D-L-3	77314024	36357530	97.37	92.96	56.18	94.05%	86.06%	8.00%
T1-B-L-1	67600914	31710582	97.27	92.56	56.32	93.82%	86.45%	7.36%
T1-B-L-2	65900486	30856463	97.09	92.21	56.26	93.65%	87.09%	6.55%
T1-B-L-3	68710068	32180876	97.11	92.27	56.21	93.67%	86.65%	7.02%
T1-C-L-1	65819144	31059541	97.21	92.44	55.24	94.38%	83.31%	11.07%
T1-C-L-2	66410548	31300365	97.22	92.48	55.4	94.26%	86.97%	7.29%
T1-C-L-3	86592230	41187513	97.56	93.13	55.62	95.13%	87.23%	7.90%
T1-D-L-1	65998152	31217355	97.45	93.06	56.6	94.60%	85.27%	9.34%
T1-D-L-2	64623480	30403432	97.06	92.14	56.75	94.09%	86.98%	7.11%
T1-D-L-3	69101260	32423503	97.17	92.44	56.75	93.84%	86.64%	7.20%
T2-A-L-1	74460748	35165230	97.47	93.27	57.66	94.45%	83.54%	10.91%
T2-A-L-2	67675478	31969735	97.34	92.93	57.7	94.48%	83.96%	10.52%
T2-A-L-3	79124390	37370014	97.37	93.06	57.57	94.46%	82.29%	12.17%
T2-B-L-1	65552724	30826515	97.36	92.91	56.67	94.05%	85.51%	8.54%
T2-B-L-2	73940060	34800738	97.24	92.44	56.89	94.13%	86.98%	7.15%
T2-B-L-3	70574386	33209521	97.36	92.94	56.97	94.11%	87.03%	7.08%
T2-C-L-1	67400870	31641359	97.38	92.91	55.56	93.89%	85.55%	8.34%
T2-C-L-2	71199134	33292183	96.96	91.97	55.54	93.52%	85.88%	7.64%
T2-C-L-3	64275602	30198599	97.3	92.7	55.57	93.97%	86.13%	7.84%
T2-D-L-1	68247848	32239432	97.81	94.27	57.19	94.48%	88.51%	5.97%
T2-D-L-2	65652762	30929274	97.9	94.45	56.78	94.22%	88.20%	6.02%
T2-D-L-3	68521852	32326872	97.56	93.32	56.38	94.35%	87.78%	6.58%

Total reads: counts of clean reads: Counts of mapped reads and the proportion of that in clean data; Unique-mapped Reads: Counts of reads mapped to a unique position on reference genome and proportion of that in clean data; Multiple mapped reads: Counts of reads mapped to multiple positions on reference genome and proportion of that in clean data; Q20: Percentage of bases with Q-score 20; Q30: Percentage of bases with Q-score 30.

Table S3. Descriptions of 62 DEGs

Funcation	Gene ID	Gene name	Description
Photosynthesis	TraesCS1A03G0988100	WHAB1.6	Chlorophyll A-B binding protein
	TraesCS1B03G1053300	WHAB1.6	Chlorophyll A-B binding protein
	TraesCS1D03G0954900	WHAB1.6	Chlorophyll A-B binding protein
	TraesCS1A03G0568300	BAM3	Beta-amylase
	TraesCS4D03G0155400	GUN4	Tetrapyrrole-binding protein
	TraesCS3B03G0355600	PPT3	Phosphoenolpyruvate/phosphate translocator
	TraesCS1A03G0745100	AGXT2.3	Alanine--glyoxylate aminotransferase
	TraesCS1D03G0712600	AGXT2.3	Alanine--glyoxylate aminotransferase
Antioxidants	TraesCS2D03G0229100	--	Peroxidase
	TraesCS3A03G0704900	RhGT1	Anthocyanidin 5,3-O-glucosyltransferase
	TraesCS7A03G1091500	--	Anthocyanidin reductase
	TraesCS7B03G0939900	--	Anthocyanidin reductase
Transcription, modifications	TraesCS1D03G0291200	TECPR1	Tectonin beta-propeller repeat-containing protein 1
	TraesCS1B03G1070300	HSFA2C	Heat stress transcription factor A-2c
	TraesCS1D03G0889300	HSFA2C	Heat stress transcription factor A-2c
	TraesCS2A03G0964500	HSFB2A	Heat stress transcription factor B-2a
	TraesCS2B03G1068900	HSFB2A	Heat stress transcription factor B-2a
	TraesCS2D03G0906200	HSFB2A	Heat stress transcription factor B-2a
	TraesCS4A03G0052300	HSFA2D	Heat stress transcription factor A-2d
	TraesCS4D03G0655500	HSFA2D	Heat stress transcription factor A-2d
	TraesCS7D03G1169400	--	Chaperone protein dnaJ
	TraesCS3B03G0293500	ZFC3H1	Zinc finger protein
	TraesCS2A03G0321700	MPSR1	E3 ubiquitin-protein ligase
	TraesCS2B03G0442100	MPSR1	E3 ubiquitin-protein ligase
	TraesCS2D03G0341300	MPSR1	E3 ubiquitin-protein ligase
	TraesCS7A03G0785600	CHIP	E3 ubiquitin-protein ligase
	TraesCS4D03G0504300	CRRSP55	Cysteine-rich repeat secretory protein
	TraesCS3B03G0790800	CLPB1	Chaperone protein
	TraesCS4A03G0004100	TMEM131	Transmembrane protein 131-like
	TraesCS6A03G1026500	--	BAG domain
	TraesCS5A03G0463500	--	HD-ZIP IV family
Signal	TraesCS1A03G0803800	SDI2	Protein SULFUR DEFICIENCY-INDUCED
	TraesCS1B03G0925400	SDI1	Protein SULFUR DEFICIENCY-INDUCED
	TraesCS1D03G0772600	SDI1	Protein SULFUR DEFICIENCY-INDUCED
	TraesCS6B03G0215700	LIS	S-(+)-linalool synthase
	TraesCS6D03G0135000	LIS	S-(+)-linalool synthase
	TraesCS3A03G0590000	At2g42960	Protein kinase domain-containing protein
	TraesCS2B03G1397000	EPHX2	Epoxide hydrolase 2
Cell wall biogenesis	TraesCS4B03G0679500	BXL7	beta-D-xylosidase
	TraesCS4D03G0606000	BXL7	beta-D-xylosidase
	TraesCS7A03G1036000	XTH23	xyloglucan endotransglucosylase/hydrolase protein
	TraesCS7B03G0876800	XTH23	xyloglucan endotransglucosylase/hydrolase protein

	TraesCS7D03G0991500	XTH25	xyloglucan endotransglucosylase/hydrolase protein
	TraesCS1D03G0989700	At5g56590	Glucan endo-1,3-beta-glucosidase
	TraesCS3D03G1048700	--	Glucan endo-1,3-beta-glucosidase
Others	TraesCS3A03G0929100	--	Late embryogenesis abundant protein
	TraesCS3D03G0343300	CYP736A117	Cytochrome P450
	TraesCS7A03G0869800	ABCG42	ABC transporter
	TraesCS4B03G0796500	DTX27	Protein DETOXIFICATION 27
	TraesCS7B03G0081700	DTX27	Protein DETOXIFICATION 27
	TraesCS2B03G0603800	APR1	5'-adenylylsulfate reductase
	TraesCS2D03G0488800	APR1	5'-adenylylsulfate reductase
	TraesCS7A03G1041500	AAE	Acetylajmalan esterase
	TraesCS1D03G0904000	--	Avr9/Cf-9 rapidly elicited protein
	TraesCS6B03G0289700	--	Response to low sulfur protein
	TraesCS7A03G0577800	--	N-acetyl-gamma-glutamyl-phosphate reductase
	TraesCS3B03G1056500	--	DUF1685 family protein
	TraesCS3D03G0863600	--	DUF1685 family protein
	Traes_newGene_10289	--	--
	Traes_newGene_34725	--	--
	Traes_newGene_62723	--	--
	Traes_newGene_63868	--	--

Table S4. Hub genes annotation

Hub gene ID	Description	Gene name	MCC score
TraesCS_newGene_8815	--	--	5181685800
TraesCS_newGene_19980	--	--	3714561720
TraesCS2D03G0495200	Protein C2-DOMAIN ABA-RELATED 8	CAR8	3580696296
TraesCS_newGene_10205	Synaptotagmin-5	SYT5	3573162408
TraesCS7B03G0849200	Cell number regulator 13	CNR13	3360559224
TraesCS3B03G0812300	Senescence regulator	--	3228583920
TraesCS1D03G0804900	Gamma-glutamyl hydrolase	GGH1	3158354928
TraesCS7B03G0604900	Gamma-glutamyl hydrolase	GGH1	3080253648
TraesCS7D03G0504000	Carboxyl-terminal-processing peptidase	CTPA3	2805193848
TraesCS4A03G0291500	Vicilin-like seed storage protein	At2g28490	2803441704
